# G3BP1 and SLU7 Jointly Promote Immune Evasion by Downregulating MHC‐I via PI3K/Akt Activation in Bladder Cancer

**DOI:** 10.1002/advs.202305922

**Published:** 2023-12-12

**Authors:** Xianchong Zheng, Jiawei Chen, Minhua Deng, Kang Ning, Yulu Peng, Zhenhua Liu, Xiangdong Li, Zhaohui Zhou, Huancheng Tang, Yaoying Li, Tiebang Kang, Zhuowei Liu

**Affiliations:** ^1^ Department of Urology Sun Yat‐sen University Cancer Center Guangzhou 510060 P. R. China; ^2^ State Key Laboratory of Oncology in South China Guangdong Provincial Clinical Research Center for Cancer Sun Yat‐sen University Cancer Center Guangzhou 510060 P. R. China; ^3^ Department of Urology Sun Yat‐sen University Cancer Center Gansu Hospital Lanzhou 730000 P. R. China; ^4^ Department of Urology Shunde Hospital Southern Medical University (The First People's Hospital of Shunde Foshan) Foshan 528000 P. R. China

**Keywords:** bladder cancer, epigallocatechin gallate, immune checkpoint inhibitors, immune evasion, major histocompatibility complex class I, Ras GTPase‐activating protein‐binding protein 1

## Abstract

Immune checkpoint inhibitors (ICIs) show promise as second‐line treatment for advanced bladder cancer (BLCA); however, their responsiveness is limited by the immune evasion mechanisms in tumor cells. This study conduct a Cox regression analysis to screen mRNA‐binding proteins and reveals an association between Ras GTPase‐activating protein‐binding protein 1 (G3BP1) and diminished effectiveness of ICI therapy in patients with advanced BLCA. Subsequent investigation demonstrates that G3BP1 enhances immune evasion in BLCA cells by downregulating major histocompatibility complex class I (MHC‐I) through phosphoinositide 3‐kinase (PI3K)/Akt signaling activation. Mechanistically, G3BP1 interacts with splicing factor synergistic lethal with U5 snRNA 7 (SLU7) to form a complex with poly(A)‐binding protein cytoplasmic 1 and eukaryotic translation initiation factor 4 gamma 1. This complex stabilizes the closed‐loop structure of the mRNAs of class IA PI3Ks and consequently facilitates their translation and stabilization, thereby activating PI3K/Akt signaling to downregulate MHC‐I. Consistently, targeting G3BP1 with epigallocatechin gallate (EGCG) impedes immune evasion and sensitizes BLCA cells to anti‐programmed cell death (PD)‐1 antibodies in mice. Thus, G3BP1 and SLU7 collaboratively contribute to immune evasion in BLCA, indicating that EGCG is a precision therapeutic agent to enhance the effectiveness of anti‐PD‐1 therapy.

## Graphical abstract

Immune checkpoint inhibitors (ICIs) show promise as second‐line treatment for advanced bladder cancer (BLCA), but the immune evasion mechanisms in tumor cells hinder their effectiveness. This study links G3BP1 to diminished ICI efficacy in advanced BLCA patients. Explore the intricate role of G3BP1 in immune evasion, its collaboration with SLU7, and the potential of epigallocatechin gallate as a precision therapeutic.

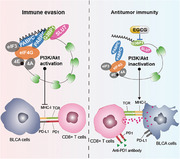

## 1 Introduction

Bladder cancer (BLCA) is a prevalent malignancy worldwide, with an annual incidence of >500 000 cases and an estimated death toll of 200 000.^[^ ^1^ ^]^ Muscle‐invasive BLCA (MIBC) is a malignant type of BLCA, with a 5‐year survival rate of only 50%.^[^ ^2^ ^]^ Cisplatin‐based chemotherapy remains the mainstay of treatment for unresectable and metastatic MIBCs, but most patients tend to relapse. Moreover, the efficacy of traditional second‐line treatment options, such as paclitaxel, is not promising. Over the past decade, immune checkpoint inhibitors (ICIs), such as monoclonal antibodies targeting programmed cell death‐1 (PD‐1) and programmed cell death‐ligand 1 (PD‐L1), have been approved for patients with advanced BLCA. Although ICI represents an important breakthrough, the response rate in BLCA remains quite limited at <30%.^[^ ^3^ ^]^

Immunosuppressive tumor microenvironment (TME) is a critical cause of tumor immune evasion and immunotherapy insensitivity, as evidenced by the exhaustion and hypoinfiltration of cytotoxic tumor‐infiltrating lymphocytes (TILs;, e.g., cluster of differentiation 8 [CD8]+ T cells).^[^ ^4^ ^]^ The first step in the recognition of tumor cells by cytotoxic CD8+ T cells is the binding of T cell receptor (TCR) to antigens presented on major histocompatibility complex class I (MHC‐I).^[^ ^5^ ^]^ However, most tumor cells, including BLCA cells, have low MHC‐I expression.^[^ ^6^ ^]^ Dysregulation of MHC‐I and other immune checkpoint signaling has enabled tumor cells to evade immune surveillance, thereby creating an immunosuppressive TME.^[^ ^7^ ^]^ Therefore, identifying the mechanisms underlying dysregulated immune checkpoint signaling in tumor cells may provide therapeutic targets to enhance the efficacy of ICIs for patients with BLCA.

The physiological functions of somatic cells depend on the homeostasis of mRNA turnover.^[^ ^8^ ^]^ An imbalance in mRNA turnover has been linked to several diseases, such as cancer and immune disorders.^[^ ^9^ ^]^ RNA‐binding proteins (RBPs) bind to specific RNAs and direct their life events, such as maturation, subcellular localization, modification, translation, and decay.^[^ ^10^ ^]^ mRNA‐binding proteins (mRBPs) are a major class of RBPs, and different mRBPs can bind to and regulate specific mRNAs through their unique RNA‐binding domains (RBDs).^[^ ^10b^ ^]^ Recent studies have revealed that mRBP dysregulation is closely associated with tumor progression.^[^ ^11^ ^]^ However, the mechanism by which mRBPs regulate BLCA immune evasion remains unclear. Therefore, we performed a Cox regression analysis to screen mRBPs that are potentially associated with immune evasion in patients who participated in the IMvigor210 cohort study,^[^ ^12^ ^]^ which investigated the efficacy of anti‐PD‐L1 antibody in patients with advanced BLCA. Among the top‐hit mRBPs, Ras GTPase‐activating protein‐binding protein 1 (G3BP1), an mRBP known for assembling stress granules,^[^ ^13^ ^]^ attracted our interest because of its unknown implication in tumor immune evasion.

Herein, we revealed that G3BP1 interacts with splicing factor synergistic lethal with U5 snRNA 7 (SLU7), an RBP known to regulate pre‐mRNA splicing and liver homeostasis,^[^ ^14^ ^]^ to stabilize the closed‐loop structure of the mRNAs of class IA PI3Ks, thereby facilitating their translation and stabilization. This leads to the activation of phosphoinositide 3‐kinase (PI3K)/Akt signaling, which in turn downregulate MHC‐I to promote BLCA immune evasion. Therefore, targeting G3BP1 with epigallocatechin gallate (EGCG) impedes immune evasion and thus sensitizes BLCA to anti‐PD‐1 antibodies.

## 2 Results

### 2.1 G3BP1 Promotes Immune Evasion by Downregulating MHC‐I in BLCA Cells

**Figure 1:**
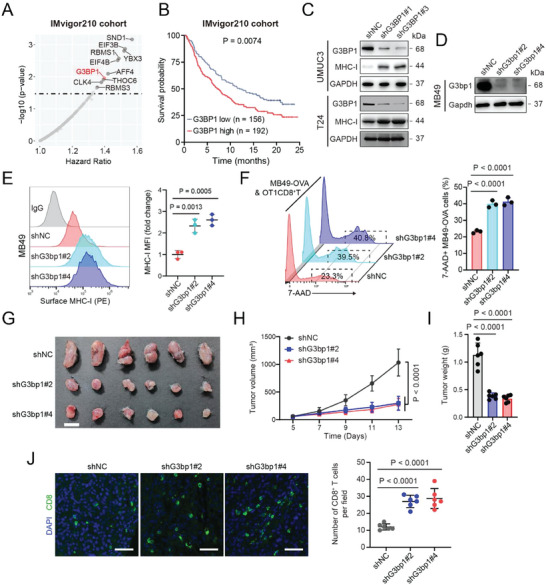
G3BP1 knockdown in BLCA cells upregulates MHC‐I and impedes immune evasion. A) The effect of mRBPs on the survival of patients with BLCA who received anti‐PD‐L1 antibody therapy was determined using Kaplan–Meier and Cox regression analyses. The top‐hit mRBPs in terms of positive correlation with the hazard ratio are presented. The mRNA expression data were derived from the IMvigor210 cohort. B) Kaplan–Meier curve constructed using the best cutoff expression of G3BP1 in the IMvigor210 cohort. C) Western blotting was used to detect the protein expression levels of G3BP1 and MHC‐I in human BLCA cell lines UMUC3 and T24 with stable G3BP1 knockdown. GAPDH was used as the internal control. D) Western blotting was used to detect the protein expression levels of G3bp1 in the murine BLCA cell line MB49 with stable G3bp1 knockdown. E) Flow cytometry was used to analyze the expression of MHC‐I on the surface of MB49 cells with stable G3bp1 knockdown. MHC‐I expression was quantified based on the mean fluorescence intensity (MFI). F) mCherry‐expressing MB49‐OVA cells with G3bp1 knockdown were cocultured with antigen‐activated CTLs for 24 h. Cytotoxicity was determined by analyzing the percentage of 7‐AAD+ cells among mCherry+ cells. G) Endpoint image of tumors from C57BL/6J mice bearing subcutaneous bladder tumors (implanted with 3 × 10^5^ MB49 cells with stable G3bp1 knockdown). Scale bar, 1 cm. H) Tumor volumes on the indicated days after inoculation with MB49 cells. I) Tumors were weighed. J) Immunofluorescent staining of CD8 in MB49 tumors, and quantification of CD8 signals. Scale bar, 50 µm. Error bars represent standard deviation (SD) (*n* = 3 in E and F; *n* = 6 in G–J). *P*‐values are presented and were calculated using the log‐rank test (B), one‐way analysis of variance (ANOVA) (E, F, I, and J), and two‐way ANOVA (H).

To investigate the role of mRBPs in immune evasion, we analyzed the effect of mRBPs on the survival of patients from the IMvigor210 cohort.^[^ ^12^ ^]^ Several top‐hit mRBPs demonstrating a positive correlation with the hazard ratio (**Figure** **1**A) have been identified as significant contributors to tumor immune evasion (e.g., SND1,^[^ ^15^ ^]^ EIF3B,^[^ ^16^ ^]^ and RBMS1^[^ ^17^ ^]^) as well as BLCA progression (e.g., YBX3,^[^ ^18^ ^]^ EIF4B,^[^ ^19^ ^]^ and AFF4^[^ ^20^ ^]^). This analysis emphasizes the crucial role of mRBPs in facilitating tumor immune evasion. Among other mRBPs, G3BP1 attracted our interest due to its unexplored role in tumor immune evasion and BLCA progression. Moreover, high G3BP1 expression was associated with unfavorable outcomes in patients from the IMvigor210 cohort (Figure 1B). G3BP1 knockdown markedly promoted the expression of HLA‐A, HLA‐B, and HLA‐C, while slightly promoting the expression of CD80, CD86, CD276, B7‐H4, PD‐L2, and GITRL in UMUC3 cells (Figure S1, Supporting Information). As HLA‐A, HLA‐B, and HLA‐C are major components of MHC‐I, we reasoned that G3BP1 may suppress MHC‐I expression to promote immune evasion in BLCA cells. This phenomenon was confirmed by the finding that G3BP1 knockdown markedly promoted MHC‐I expression in both UMUC3 and T24 cells (Figure 1C). Likewise, G3bp1 knockdown increased the cell surface MHC‐I expression in murine BLCA cell line MB49, as demonstrated via flow cytometry (Figure 1D,E).

Tumor‐cell surface MHC‐I is essential for the recognition of tumor cells by cytotoxic T lymphocytes (CTLs);^[^ ^5^ ^]^ therefore, we assessed the effect of G3BP1 on immune evasion. The in vitro T cell killing assay revealed that G3bp1 knockdown in MB49‐ovalbumin (OVA) cells increased the killing capacity of OT1CD8^+^ T cells (Figure 1F). Moreover, in vivo G3bp1 knockdown in MB49 cells decreased the tumor volume and weight and enhanced the infiltration of CD8+ T cells into tumor (Figure 1G–J). These results indicate that G3BP1 downregulates MHC‐I in BLCA cells to promote immune evasion.

### 2.2 G3BP1 Downregulates MHC‐I to Promote Immune Evasion by Activating PI3K/Akt Signaling in BLCA Cells

**Figure 2:**
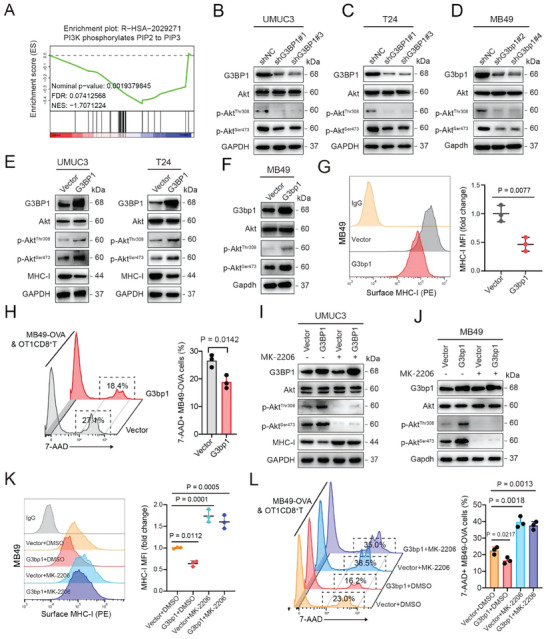
G3BP1 downregulates MHC‐I and promotes immune evasion by activating PI3K/Akt. A) UMUC3 cells with G3BP1 knockdown were subjected to RNA‐seq analysis. GSEA plot showing the correlation between G3BP1 expression and the PI3K signaling pathway based on RNA‐seq data. B–D) Western blotting was used to detect the protein expression levels of G3BP1, total Akt, p‐Akt^Thr308^, and p‐Akt^Ser473^ in UMUC3, T24, and MB49 cells with stable G3BP1 knockdown. GAPDH was used as the internal control. E and F) Western blotting was used to detect the protein expression levels of G3BP1, total Akt, p‐Akt^Thr308^, p‐Akt^Ser473^, and/or MHC‐I in UMUC3, T24, and MB49 cells with stable G3BP1 overexpression. G) Flow cytometry was used to analyze the expression of MHC‐I on the surface of MB49 cells in (F). MHC‐I expression was quantified based on the mean fluorescence intensity (MFI). H) mCherry‐expressing MB49‐OVA cells with G3bp1 overexpression were cocultured with antigen‐activated CTLs for 24 h. Cytotoxicity was determined by analyzing the percentage of 7‐AAD+ cells among mCherry+ cells. I,J) G3BP1‐overexpressing UMUC3 and MB49 cells were treated with or without 1.0 µm MK‐2206 for 24 h, and western blotting was used to determine the protein expression levels of G3BP1, total Akt, p‐Akt^Thr308^, p‐Akt^Ser473^, and/or MHC‐I. K) Flow cytometry was used to analyze MHC‐I expression on the surface of MB49 cells in (J). MHC‐I expression was quantified based on MFI. L) mCherry‐expressing MB49‐OVA cells with or without G3bp1 overexpression and MK‐2206 treatment were cocultured with antigen‐activated CTLs for 24 h. Cytotoxicity was determined by analyzing the percentage of 7‐AAD+ cells among mCherry+ cells. Error bars represent SD (*n* = 3 in G, H, K, and L). *P*‐values are presented and were calculated using unpaired, two‐tailed Student's *t*‐test (G and H), and one‐way ANOVA (K and L).

To determine the mechanism by which G3BP1 inhibits MHC‐I expression, we performed RNA sequencing (RNA‐seq) in UMUC3 cells with G3BP1 knockdown. Kyoto Encyclopedia of Genes and Genomes (KEGG) pathway enrichment analysis and gene set enrichment analysis (GSEA) demonstrated that G3BP1 knockdown inhibited the PI3K/Akt signaling pathway (Figure S2, Supporting Information; **Figure** **2**A), which is known to repress tumor immunogenicity by downregulating MHC‐I.^[^ ^21^ ^]^ Indeed, G3BP1 knockdown decreased p‐Akt^Thr308^ and p‐Akt^Ser473^ expression (PI3K effectors), but not total Akt expression, in UMUC3, T24, and MB49 cells (Figure 2B–D). Consistently, G3BP1 overexpression in both human and murine BLCA cells upregulated p‐Akt^Thr308^ and p‐Akt^Ser473^ and downregulated MHC‐I (Figure 2E–G). Further, G3bp1 overexpression in MB49‐OVA cells decreased the killing capacity of OT1CD8^+^ T cells (Figure 2H). As expected, Akt inhibition by MK‐2206 upregulated MHC‐I in both UMUC3 and MB49 cells and impeded immune evasion in MB49 cells (Figure S3, Supporting Information). Moreover, MK‐2206 restored the effects of G3BP1 overexpression on p‐Akt^Thr308^, p‐Akt^Ser473^, and MHC‐I expression as well as the killing capacity of OT1CD8^+^ T cells in BLCA cells (Figure 2I–L). These results indicate that G3BP1 downregulates MHC‐I to promote immune evasion by activating PI3K/Akt signaling in BLCA cells.

### 2.3 G3BP1 Increases Both mRNA and Protein Levels of Class IA PI3Ks via Binding to Their mRNAs in BLCA Cells

**Figure 3:**
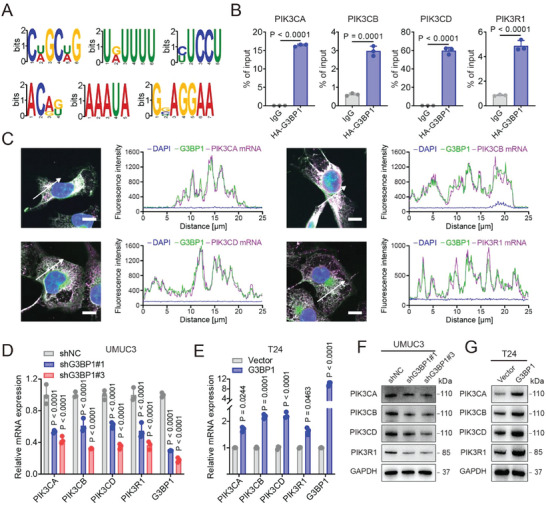
G3BP1 binds to the mRNAs of class IA PI3Ks and promotes their expression. A) Cell lysates from UMUC3 cells with stable expression of HA‐G3BP1 were immunoprecipitated with anti‐HA antibody, and the purified RNA from the immunoprecipitants was subjected to RNA‐seq analysis. Motif spacing analysis was used to identify the significant RNA motifs of HA‐G3BP1 on RNAs. B) The RIP assay with anti‐HA antibodies was performed using UMUC3 cells with stable expression of HA‐G3BP1. qRT–PCR analysis was used to evaluate the mRNA levels of class IA PI3Ks in the immunoprecipitants. C) The locations of G3BP1 and the mRNAs of class IA PI3Ks in UMUC3 cells were analyzed using immuno‐FISH. Scale bar, 10 µm. The fluorescence intensity curves of DAPI (blue), G3BP1 (green), and mRNA (purple) that crossed the arrows were analyzed. D and E) qRT–PCR analysis was used to evaluate the mRNA levels of G3BP1 and the class IA PI3Ks in UMUC3 and T24 cells with G3BP1 knockdown or overexpression. F,G) Western blotting was used to detect the protein expression levels of class IA PI3Ks in UMUC3 and T24 cells with G3BP1 knockdown or overexpression. GAPDH was used as the internal control. Error bars represent SD (*n* = 3 in B, D, and E). *P*‐values are presented and were calculated using unpaired two‐tailed Student's *t*‐test (B) and two‐way ANOVA (D and E).

To investigate the mechanism by which G3BP1 activates PI3K/Akt signaling, we performed RNA immunoprecipitation sequencing (RIP‐seq) using UMUC3 cells that stably express HA‐G3BP1. Notably, we revealed that G3BP1 binds to the mRNAs of class IA PI3Ks (PIK3CA, PIK3CB, PIK3CD, and PIK3R1) through multiple motifs (**Figure** **3**A; Figure S4, Supporting Information), which were verified via the RIP assay (Figure 3B). The colocalization of G3BP1 with the mRNA of PIK3CA, PIK3CB, PIK3CD, or PIK3R1 in UMUC3 cells was visualized via immunofluorescence in situ hybridization (immuno‐FISH) using an antibody against G3BP1 and specific probes for PIK3CA, PIK3CB, PIK3CD or PIK3R1 mRNA (Figure 3C). Furthermore, we revealed that G3BP1 knockdown and overexpression decreased and increased both mRNA and protein levels of PIK3CA, PIK3CB, PIK3CD, and PIK3R1 in cells, respectively (Figure 3D–G). These results indicate that G3BP1 binds to the mRNAs of class IA PI3Ks to increase their mRNA and protein levels.

### 2.4 SLU7 Interacts with G3BP1 and is Required for the G3BP1‐Dependent Regulation of PI3K/Akt/MHC‐I Signaling

**Figure 4:**
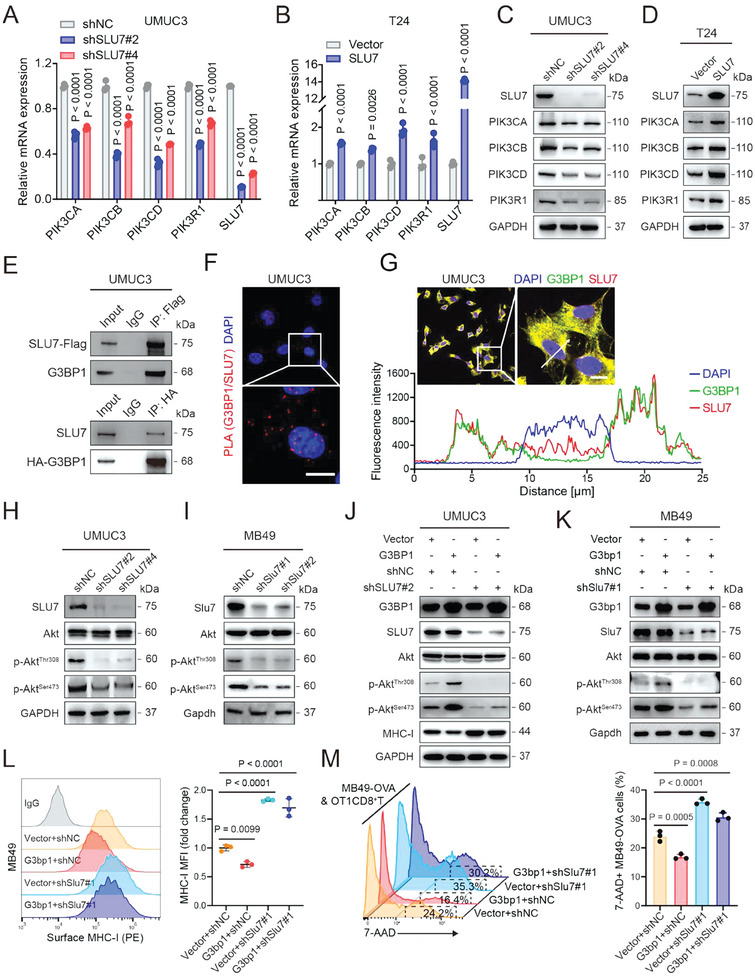
G3BP1 interacts with SLU7 to promote PI3K/Akt signaling activation, downregulating MHC‐I and promoting immune evasion. A,B) qRT–PCR analysis was used to evaluate the mRNA levels of SLU7 and the class IA PI3Ks in UMUC3 and T24 cells with SLU7 knockdown or overexpression. C,D) Western blotting was used to detect the protein expression levels of class IA PI3Ks in UMUC3 and T24 cells with SLU7 knockdown or overexpression. E) Cell lysates from UMUC3 cells with stable expression of SLU7‐Flag or HA‐G3BP1 were immunoprecipitated with anti‐Flag or anti‐HA antibodies, and immunoblotting was conducted with the indicated antibodies. F) The PLA was used to evaluate the interaction between G3BP1 and SLU7 in UMUC3 cells. Scale bar, 10 µm. G) IF was used to analyze the locations of G3BP1 and SLU7 in UMUC3 cells. Scale bar, 10 µm. Fluorescence intensity curves of DAPI (blue), G3BP1 (green), and SLU7 (red) that crossed the arrow were analyzed. H,I) The protein expression levels of SLU7, total Akt, p‐Akt^Thr308^, and p‐Akt^Ser473^ in UMUC3 and MB49 cells with stable SLU7 knockdown were detected using western blotting. J,K) Western blotting was used to detect the protein expression levels of G3BP1, SLU7, total Akt, p‐Akt^Thr308^, p‐Akt^Ser473^, and/or MHC‐I in G3BP1‐overexpressing UMUC3 and MB49 cells with SLU7 knockdown. L) Flow cytometry was used to analyze the expression of MHC‐I on the surface of MB49 cells in (K). MHC‐I expression was quantified based on the mean fluorescence intensity (MFI). M) mCherry‐expressing MB49‐OVA cells with or without G3bp1 overexpression and Slu7 knockdown were cocultured with antigen‐activated CTLs for 24 h. Cytotoxicity was determined by analyzing the percentage of 7‐AAD+ cells among mCherry+ cells. Error bars represent SD (*n* = 3 in A, B, L, and M). *P*‐values are presented and were calculated using two‐way ANOVA (A and B) and one‐way ANOVA (L and M).

G3BP1 regulates downstream signaling by interacting with different proteins, especially RBPs;^[^ ^22^ ^]^ therefore, we hypothesized that G3BP1 similarly activates PI3K/Akt signaling. Accordingly, we used coimmunoprecipitation (co‐IP) and mass spectrometry (MS) to identify G3BP1‐interacting proteins in UMUC3 cells (Figure S5A, Supporting Information). We identified 213 RBPs that interacted with G3BP1 (Figure S5B, Supporting Information). SLU7, one of the top hits among these G3BP1‐interacting RBPs (Figure S5C, Supporting Information), attracted our attention. This is because the overpression of SLU7, but not other top‐hit RBPs, increased p‐Akt^Ser473^ expression in UMUC3 cells (Figure S5D,E, Supporting Information). Similar to G3BP1, high SLU7 expression was associated with unfavorable outcomes in patients from the IMvigor210 cohort (Figure S6, Supporting Information). Indeed, the binding of SLU7 to mRNAs of class IA PI3Ks was verified via the RIP assay using UMUC3 cells that express SLU7‐Flag (Figure S7, Supporting Information). Further, SLU7 knockdown and overexpression decreased and increased both mRNA and protein levels of class IA PI3Ks, respectively (**Figure** **4**A–D). These results indicate that SLU7 increases both mRNA and protein levels of class IA PI3Ks by binding to their mRNAs in BLCA cells.

Moreover, ectopic SLU7 and G3BP1 formed a complex with endogenous G3BP1 and SLU7 in cells, respectively (Figure 4E; Figure S8A, Supporting Information), and the proximity ligation assay (PLA) and immunofluorescence (IF) assay using UMUC3 and T24 cells were performed to confirm the interaction of endogenous G3BP1 and SLU7 (Figure 4F,G; Figure S8B,C, Supporting Information). SLU7 knockdown decreased the expression of p‐Akt^Thr308^ and p‐Akt^Ser473^ (Figure 4H,I) and impaired the effect of G3BP1 overexpression on p‐Akt^Thr308^, p‐Akt^Ser473^, and MHC‐I expression as well as the killing capacity of OT1CD8^+^ T cells (Figure 4J–M). These results revealed that SLU7 may interact with G3BP1 and is required for the G3BP1‐dependent regulation of the PI3K/Akt/MHC‐I axis in BLCA cells to promote immune evasion.

Interestingly, G3BP1 and SLU7 knockdown substantially reduced the binding of SLU7 and G3BP1 to the mRNAs of class IA PI3Ks in cells, respectively, as demonstrated via the RIP assay (Figure S9A–D, Supporting Information). This was further verified via immuno‐FISH analysis, revealing that G3BP1 and SLU7 knockdown disrupted the colocalization of SLU7 and G3BP1, respectively, with the mRNAs of class IA PI3Ks (Figure S9E, Supporting Information). Taken together, these findings indicate that the G3BP1‐SLU7 complex binds to the mRNAs of class IA PI3Ks in a G3BP1‐ and SLU7‐dependent manner.

### 2.5 G3BP1 Interacts with SLU7 to Stabilize the Poly(A) Binding Protein Cytoplasmic 1 (PABPC1)‐Mediated Closed‐Loop Structure of the mRNAs of Class IA PI3Ks

**Figure 5:**
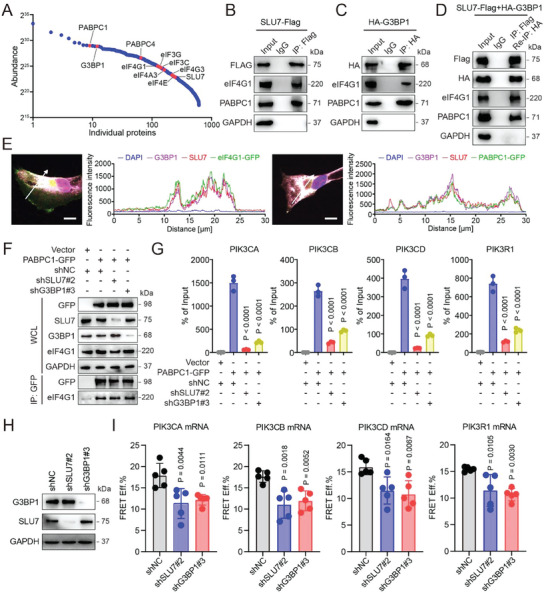
G3BP1 and SLU7 form a complex with PABPC1 and eIF4G1 to stabilize the closed‐loop structure of the mRNAs of class IA PI3Ks. A) Scatter plot of the G3BP1‐interacting proteins identified using MS. B and C) Cell lysates from UMUC3 cells with stable expression of SLU7‐Flag or HA‐G3BP1 were immunoprecipitated with anti‐Flag or anti‐HA antibodies, and immunoblotting was performed with the indicated antibodies. D) Cell lysates from UMUC3 cells with simultaneous stable expression of SLU7‐Flag and HA‐G3BP1 were primarily immunoprecipitated with anti‐Flag antibody and then blocked with Flag peptide. The resultant immunoprecipitants were subjected to re‐IP with anti‐HA antibodies and immunoblotted with the indicated antibodies. E) IF was used to analyze the locations of eIF4G1 or PABPC1 with SLU7 and G3BP1 in UMUC3 cells stably expressing eIF4G1‐GFP or PABPC1‐GFP. Scale bar, 10 µm. The fluorescence intensity curves of DAPI (blue), eIF4G1‐GFP (green), PABPC1‐GFP (green), SLU7 (red), and G3BP1 (purple) that crossed the arrows were analyzed. F) Cell lysates from UMUC3 cells with PABPC1‐GFP stable expression and SLU7 or G3BP1 knockdown were immunoprecipitated with anti‐GFP antibody. Western blotting was used to detect the expression levels of PABPC1‐GFP, SLU7, G3BP1, and eIF4G1 in whole cell lysates and immunoprecipitants. G) qRT–PCR analysis was used to evaluate the mRNA expression levels of class IA PI3Ks in the immunoprecipitants. H) Western blotting was used to detect the expression levels of G3BP1 and SLU7 in UMUC3 cells with SLU7 knockdown or G3BP1 knockdown. I) FISH–FRET was performed in UMUC3 cells with SLU7 or G3BP1 knockdown using Cy3‐labeled 5′‐UTR probes and Cy5‐labeled 3′‐UTR probes targeting the mRNAs of PI3Ks. The mean values of FRET efficiency are shown. Error bars represent SD (*n* = 3 in G; *n* = 5 in I). *P*‐values are presented and were calculated using one‐way ANOVA (G and I).

The G3BP1 interactome was established to elucidate the mechanism by which the G3BP1‐SLU7 complex affects the mRNAs of class IA PI3Ks (**Figure** **5**A). Notably, in addition to SLU7, PABPCs and various eukaryotic transcription initiation factors (e.g., eIF3s and eIF4s), which are known to constitute the closed‐loop structure of mRNA,^[^ ^10^, ^23^ ^]^ were listed (Figure 5A). We then hypothesized the involvement of the G3BP1‐SLU7 complex in stabilizing the PABPC‐mediated closed‐loop structure because G3BP1 frequently binds to the 3′‐UTR (Figure S4, Supporting Information). Indeed, both G3BP1 and SLU7 interacted with PABPC1 and eukaryotic translation initiation factor 4 gamma 1 (eIF4G1) (Figure 5B,C). These proteins formed a complex, as demonstrated by the reimmunoprecipitation (re‐IP) assay (Figure 5D), and PABPC1‐GFP or eIF4G1‐GFP colocalized with both G3BP1 and SLU7 in UMUC3 cells, as demonstrated via the IF assay (Figure 5E). The interaction of G3BP1‐SLU7 with PABPC1 and eIF4G1 is dependent on G3BP1, but not on the RBDs of G3BP1 or SLU7, in cells (Figures S10 and S11, Supporting Information). Either G3BP1 or SLU7 knockdown diminishes the binding of PABPC1 to mRNAs of class IA PI3Ks, but not to eIF4G1, in cells (Figure 5F,G). These results indicate that both G3BP1 and SLU7 form a complex with PABPC1 and eIF4G1 to potentially stabilize the closed‐loop structure of the mRNAs of class IA PI3Ks.

The direct method to visualize the closed‐loop structure of mRNA in situ has not been well established. Therefore, we set up a FISH–fluorescence resonance energy transfer (FRET) method by labeling the cap and tail of mRNA using Cy3‐conjugated donor primers and Cy5‐conjugated receptor primers, respectively, and evaluated the closed‐loop structure according to the FRET efficiency between Cy3 and Cy5 (Figure S12A, Supporting Information). This FISH–FRET method was proven to be effective as knockdown of PABPC1, which is indispensable in the closed‐loop structure of mRNAs,^[^ ^10a^ ^]^ decreased the FRET efficiency of the paired dyes labeled in the mRNAs of class IA PI3Ks (Figure S12B,C, Supporting Information), indicating that PABPC1 knockdown impaired the closed‐loop structure of the mRNAs of class IA PI3Ks. Importantly, the FRET efficiency on the mRNAs of class IA PI3Ks decreased in UMUC3 cells with either G3BP1 or SLU7 knockdown (Figure 5H,I), indicating that both G3BP1 and SLU7 are required for stabilizing the closed‐loop structure of the mRNAs of class IA PI3Ks.

### 2.6 G3BP1 and SLU7 Facilitate the Translation and mRNA Stabilization of Class IA PI3Ks

**Figure 6:**
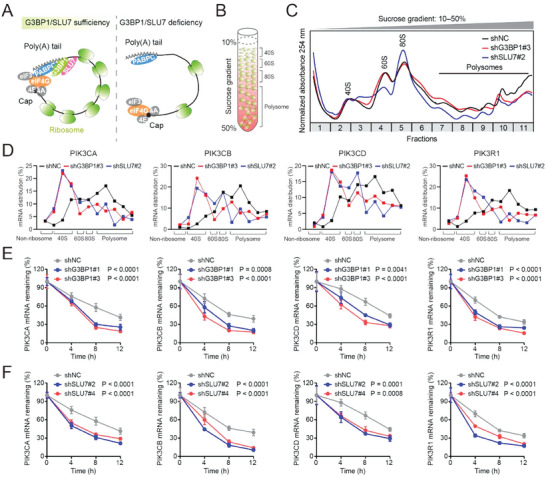
G3BP1 and SLU7 are essential for the translation and mRNA stabilization of class IA PI3Ks. A) A graphical model illustrates the mechanism by which G3BP1‐SLU7 stabilizes the closed‐loop structure and ribosome binding of the mRNAs of class IA PI3Ks. B) A graphical model illustrating the sucrose gradient distribution of subunits of ribosomes and polysomes. C) Lysates from UMUC3 cells with or without G3BP1 or SLU7 knockdown were separated using 10%–50% sucrose gradients. Polysome profiles of the gradients were generated by measuring absorbance at 254 nm. D) qRT–PCR analysis was used to evaluate the mRNA levels of class IA PI3Ks in each sucrose fraction. The mRNA distribution in each sucrose fraction was determined by normalizing the mRNA expression to that of GAPDH. E,F) UMUC3 cells with G3BP1 or SLU7 knockdown were treated with actinomycin D, and total RNA was extracted at the indicated time points. qRT–PCR analysis was used to evaluate the mRNA levels in each treatment group. The expression of mRNAs remaining at different time points was determined by normalizing the mRNA expression to that detected at 0 h. Error bars represent SD (*n* = 3 in E and F). *P*‐values are presented and were calculated using two‐way ANOVA (E and F).

The closed‐loop structure of mRNAs is crucial for maintaining translation, as evidenced by the fact that mRNAs are usually accompanied with enriched polysomes.^[^ ^10^, ^24^ ^]^ Therefore, we hypothesized that G3BP1 and SLU7 affect the translational activity of class IA PI3Ks (**Figure** **6**A). Polysome profiling analysis revealed that either G3BP1 or SLU7 knockdown reduced the proportion of class IA PI3K mRNAs distributed to polysomes in cells (Figure 6B–D), indicating that G3BP1 and SLU7 are critical for the translation of class IA PI3Ks.

Eukaryotic cells possess well‐established mechanisms of mRNA metabolism, and mRNAs with incomplete closed‐loop structures are usually susceptible to degradation.^[^ ^10a^ ^]^ Therefore, we examined the effect of G3BP1 or SLU7 on the mRNA stability of class IA PI3Ks. G3BP1 or SLU7 knockdown markedly decreased the mRNA stability of PIK3CA, PIK3CB, PIK3CD, and PIK3R1 (Figure 6E,F). These results indicate that G3BP1 and SLU7 stabilize the mRNAs of class IA PI3Ks.

### 2.7 High G3BP1 and SLU7 Expressions Correlate with BLCA Progression and Unfavorable Outcomes in Patients with BLCA

**Figure 7:**
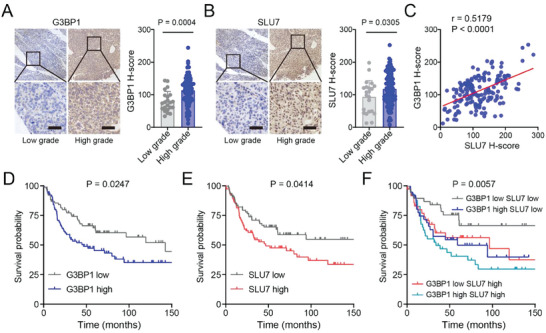
Higher expression levels of G3BP1 and SLU7 correlate with BLCA progression. A,B) IHC was used to detect the expression levels of G3BP1 and SLU7 in BLCA tumors. Images representing the staining of G3BP1 and SLU7 from low‐grade and high‐grade tumors. Scale bar, 50 µm. The H‐scores of G3BP1 and SLU7 were quantified. Error bars represent SD (*n* = 22 in “Low grade”; *n* = 122 in “High grade”). *P‐*values were calculated using unpaired two‐tailed Student's *t*‐test. C) Pearson's correlation test was used to analyze correlations between the H‐scores of G3BP1 and SLU7 in BLCA tumors; *n* = 151. D–F) Kaplan–Meier analysis and log‐rank test were used to evaluate the impact of G3BP1 and SLU7 on the survival of patients with BLCA, with the corresponding *P*‐values. The G3BP1 and SLU7 expression data used for survival analysis comprised the H‐scores described in A and B; *n* = 74 in “G3BP1 low;” *n* = 77 in “G3BP1 high;” *n* = 74 in “SLU7 low;” *n* = 77 in “SLU7 high;” *n* = 44 in “SLU7 low, G3BP1 low;” *n* = 30 in “SLU7 low, G3BP1 high;” *n* = 30 in “SLU7 high, G3BP1 low;” and *n* = 47 in “SLU7 high, G3BP1 high.”.

Next, we assessed the clinical relevance of both G3BP1 and SLU7 using BLCA samples. The protein levels of G3BP1 and SLU7 demonstrated a positive correlation, and higher protein levels of G3BP1 or SLU7 were associated with higher grades of BLCA, indicative of an unfavorable outcome in patients with BLCA (**Figure** **7**A–E). Notably, patients with BLCA showing high or low protein levels of both G3BP1 and SLU7 demonstrated the worst or best outcomes, respectively (Figure 7F). Altogether, these results indicate that high protein levels of G3BP1 and SLU7 correlate with BLCA progression and unfavorable outcomes in patients with BLCA.

### 2.8 Targeting G3BP1 with EGCG Impedes Immune Evasion and Increases the Sensitivity of Anti‐PD‐1 Antibody

**Figure 8:**
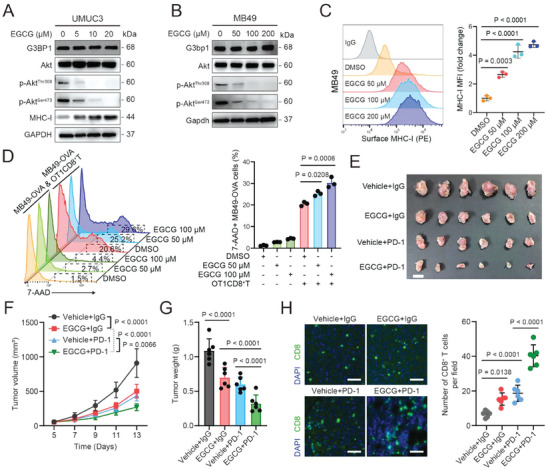
Targeting G3BP1 with EGCG impedes immune evasion and increases the sensitivity of anti‐PD‐1 antibody. A,B) UMUC3 and MB49 cells were treated with EGCG with the indicated concentrations for 48 h, and western blotting was used to detect the expression levels of G3BP1, total Akt, p‐Akt^Thr308^, p‐Akt^Ser473^, and/or MHC‐I. C) Flow cytometry was used to analyze the expression of MHC‐I on the surface of MB49 cells in (B). MHC‐I expression was quantified based on the mean fluorescence intensity (MFI). D) mCherry‐expressing MB49‐OVA cells with or without EGCG treatment were cocultured with or without antigen‐activated CTLs for 24 h. Cytotoxicity was determined by analyzing the percentage of 7‐AAD+ cells among mCherry+ cells. E–H) C57BL/6J mice bearing subcutaneous bladder tumors (implanted with 3 × 10^5^ MB49 cells) received EGCG, vehicle, IgG, or anti‐PD‐1 antibody therapy. E) Endpoint image of MB49 tumors from mice that were treated as indicated. Scale bar, 1 cm. F) Tumor volumes on the indicated days after inoculation with MB49 cells. G) Tumors were weighed. H) Immunofluorescent staining of CD8 in MB49 tumors, and quantification of CD8 signals. Scale bar, 50 µm. Error bars represent SD (*n* = 3 in C and D; *n* = 6 in E–H). *P*‐values are presented and were calculated using one‐way ANOVA (C, D, G, and H) and two‐way ANOVA (F).

EGCG, a polyphenol isolated from tea leaves, inhibits G3BP1 by binding to endogenous G3BP1 in tumor cells.^[^ ^25^ ^]^ Therefore, we investigated the potential use of EGCG to treat patients with BLCA, as EGCG has previously been used in patients with other cancer types.^[^ ^26^ ^]^ As expected, EGCG downregulated p‐Akt^Thr308^ and p‐Akt^Ser473^ and upregulated MHC‐I in a dose‐dependent manner in both UMUC3 and MB49 cells (**Figure** **8**A–C). It also increased the killing capacity of OT1CD8^+^ T cells in MB49‐OVA cells (Figure 8D). More importantly, in the MB49 tumor‐bearing immunocompetent C57BL/6J mouse model (Figure S13A, Supporting Information), the combination of EGCG with anti‐PD‐1 antibody was more efficient in inhibiting tumor growth and increasing the infiltration of CD8+ T cells into tumor than either EGCG or anti‐PD‐1 antibody alone (Figure 8E–H; Figure S13B, Supporting Information). Collectively, these results indicate that EGCG promotes MHC‐I expression to impede immune evasion and increase the sensitivity of anti‐PD‐1 antibodies by inhibiting G3BP1‐SLU7/PI3K/Akt signaling.

## 3 Discussion

**Figure 9:**
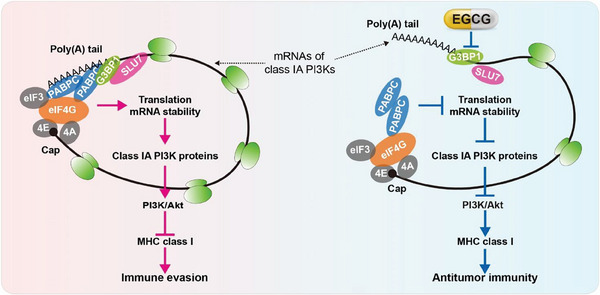
The proposed model in this study. The G3BP1‐SLU7 complex interacts with PABPC1 and eIF4G1 to stabilize the closed‐loop structure of the mRNAs of class IA PI3Ks, thereby facilitating their translation and stabilization and activating the PI3K/Akt axis to downregulate MHC‐I and promote immune evasion. In contrast, targeting G3BP1 with EGCG disrupts the PABPC1‐mediated closed‐loop structure of the mRNAs of class IA PI3Ks and eventually promotes antitumor immunity.

The dysregulated immune checkpoint signaling in tumor cells is a critical factor in the low responsiveness of ICIs for advanced BLCA. However, the mechanisms underlying immune checkpoint signaling dysregulation in tumor cells remain largely unknown. Herein, we revealed that G3BP1 downregulates MHC‐I to promote BLCA immune evasion by activating PI3K/Akt signaling. G3BP1 interacts with SLU7 to stabilize the closed‐loop structure of the mRNAs of class IA PI3Ks, thereby facilitating their translation and stabilization. Therefore, targeting G3BP1 with EGCG enhances the efficacy of anti‐PD‐1 antibody for BLCA (**Figure** **9**).

During the induction of antitumor immunity by cytotoxic CD8+ T cells, the first step in immune killing is antigen presentation by MHC‐I on tumor cells to the TCR of CD8+ T cells. However, frequent MHC‐I deficiency in most tumors,^[^ ^6^ ^]^ including BLCA, hinders the recognition of tumor cells by CTLs, ultimately resulting in tumor immune evasion and ICI insensitivity. PI3K/Akt signaling activation has recently been shown to cause MHC‐I deficiency in tumor cells,^[^ ^21^ ^]^ which was also observed in case of BLCA cells in this study. Moreover, G3BP1 downregulates MHC‐I by activating PI3K/Akt signaling to promote immune evasion of BLCA, which is a novel function of G3BP1, although G3BP1 has previously been shown to enhance antiviral immunity^[^ ^27^ ^]^ and autoimmune response.^[^ ^25^, ^28^ ^]^

Three classes of PI3Ks exist, but only class I PI3Ks have been associated with carcinogenesis and BLCA.^[^ ^29^ ^]^ The class IA PI3Ks are heterodimers, including p110 catalytic subunits (p110α/PIK3CA, p110β/PIK3CB, and p110δ/PIK3CD) and p85 regulatory subunits (p85α/PIK3R1 and p85β/PIK3R2).^[^ ^29^, ^30^ ^]^ We revealed that G3BP1 and SLU7 jointly bind to the mRNAs of most class IA PI3Ks, stabilize the mRNAs, and enhance their translation. These findings are surprising, as previous studies have revealed that G3BP1 promotes mRNA degradation by binding to UPF1 and that G3BP1 binds to the rG4 structure to maintain mRNA stability but inhibit translation.^[^ ^22^, ^31^ ^]^ This discrepancy may be because G3BP1 coordinates with SLU7 to form a complex with PABPC1 and eIF4G1 to stabilize the closed‐loop structure of the mRNAs of class IA PI3Ks,^[^ ^10^, ^23^ ^]^ indicating the new role of SLU7 in sustaining PI3K/Akt signaling to downregulate MHC‐I in BLCA cells.

SLU7 is predominantly located in the nucleus,^[^ ^14c^ ^]^ although a minor proportion is also detected in the cytoplasm, particularly after exposure to ultraviolet‐C stress.^[^ ^32^ ^]^ However, the role of SLU7 in the cytoplasm remains poorly understood. This study identified an interaction between SLU7 and G3BP1 in BLCA cells, which is consistent with the finding of a previous MS analysis conducted in hepatoma cells.^[^ ^33^ ^]^ The SLU7 interactome analysis revealed the presence of cytoplasmic proteins^[^ ^33^ ^]^ such as G3BP1 as well as other proteins responsible for preserving the closed‐loop structure of mRNAs (e.g., PABPCs, eIF3s, and eIF4s).^[^ ^10^, ^23^ ^]^ Consequently, Slu7 may enhance Akt activation in the mouse liver^[^ ^14b^ ^]^ through our proposed mechanism, although further investigation is warranted to validate this hypothesis. Thus, this study provides novel insights into the cytoplasmic role of SLU7, particularly in co‐sustaining PI3K/Akt signaling with G3BP1 in BLCA cells.

The therapeutic upregulation of MHC‐I has recently been shown to enhance the sensitivity of ICIs.^[^ ^34^ ^]^ However, as MHC‐I is also expressed in nontumor cells, an increase in MHC‐I expression in vivo may cause autoimmune disorders.^[^ ^35^ ^]^ Therefore, inhibition of G3BP1, a target present at high levels in BLCA cells, may be suitable for enhancing MHC‐I expression in BLCA cells. Indeed, inhibition of G3BP1 by EGCG notably inactivates PI3K/Akt signaling to upregulate MHC‐I in human and murine BLCA cells and suppresses the immune evasion of murine BLCA cells in vitro and in vivo. Importantly, we revealed that EGCG enhanced the efficacy of anti‐PD‐1 antibodies in murine BLCA tumors. High G3BP1 levels are associated with a poor prognosis in patients with BLCA treated with anti‐PD‐L1 antibody; thus, the use of EGCG may also enhance the efficacy of ICIs in human BLCA. To date, >100 clinical trials related to EGCG have been registered worldwide (https://clinicaltrials.gov/), including phase 3 and phase 4 clinical trials. However, no clinical trials have yet investigated the efficacy of EGCG in combination with ICIs for cancer (especially advanced BLCA). Therefore, we highly recommend that clinical trials should be conducted to investigate whether EGCG enhances the efficacy of ICIs (e.g., anti‐PD‐1 antibody) in patients with advanced BLCA.

In conclusion, G3BP1 interacts with SLU7 to form a complex with PABPC1 and eIF4G1, which stabilizes the closed‐loop structure of class IA PI3K mRNAs and facilitates their translation and stabilization, thereby activating PI3K/Akt signaling to promote immune evasion by suppressing MHC‐I expression. Therefore, targeting G3BP1 with EGCG impedes immune evasion and sensitizes BLCA cells to anti‐PD‐1 antibodies in mice. Thus, this study indicates the potential of EGCG as a precision therapeutic agent to enhance the effectiveness of anti‐PD‐1 therapy for BLCA.

## 4 Experimental Section

### Ethics Approval and Consent to Participate

The use of human tissues in this study was reviewed and approved by the Committee for Ethical Review of Research Involving Human Subjects of Sun Yat‐sen University Cancer Center (Guangzhou, China; Registration ID: B2022‐350‐01). Informed consent was obtained from all patients. All animal‐related procedures were approved by the Animal Research Committee of Sun Yat‐sen University Cancer Center (Guangzhou, China; Registration ID: L102042021040I).

### Patients and Tissue Specimens

One hunded fifty‐one formalin‐fixed and paraffin‐embedded BLCA specimens were otained from patients who underwent radical resection for BLCA at Sun Yat‐sen University Cancer Center from 2008 to 2016. None of the patients had received any antitumor therapy prior to biopsy. All samples were pathologically confirmed by two experienced pathologists using hematoxylin and eosin staining.

### Cell Lines and Cell Culture

BLCA cell lines, including T24 (human), UMUC3 (human), MB49 (murine), and human embryonic kidney 293T (HEK293T) cells, were obtained from the American Type Culture Collection (Manassas, VA, USA). T24 and MB49 cells were cultured in Roswell Park Memorial Institute (RPMI) 1640 (Invitrogen, Carlsbad, CA, USA), UMUC3 cells were cultured in minimum essential medium (containing nonessential amino acids; Invitrogen), and HEK293T cells were cultured in Dulbecco's modified Eagle medium (Invitrogen). All media were supplemented with 10% fetal bovine serum (FBS; Invitrogen) and 1% penicillin/streptomycin. The cells were incubated at 37°C under 5% CO_2_. All cell lines used in this study were authenticated using short tandem repeat profiling analysis and tested negative for mycoplasma contaminations.

### Plasmid Construction

HA‐tagged human or mouse G3BP1, Flag‐tagged human or mouse SLU7, and their truncations were cloned into the PSIN‐EF1α‐blasticidin vector. Further, Flag‐tagged LARP1, DDX21, DHX9, PHAX, RBM22, EIF2S3, FXR1, SERBP1, and LARP4 as well as GFP‐tagged PABPC1 and eIF4G1 were cloned into the PSIN‐EF1α‐blasticidin vector. Next, OVA was cloned into the CV547‐IRES‐mCherry vector. Then, shRNAs targeting human or mouse G3BP1, SLU7, and PABPC1 were cloned into the PLKO.1‐puro vector. The target sequences of shRNAs are presented in Table S1 (Supporting Information).

### Establishment of Stable Expression Cell Lines

Stable expression cell lines were established for T24, UMUC3, and MB49 by infecting them with lentiviruses (MOI = 30), followed by selection for 1 week using 0.75 µg mL^−1^ puromycin and/or 5 µg mL^−1^ blasticidin. For T cell killing assay, MB49 cells were infected with OVA‐mCherry lentivirus (MOI = 30), and mCherry‐positive single‐cell clones were selected to establish stable mCherry‐expressing MB49‐OVA cell lines. Stable MB49‐OVA cell lines with modifications using shNC, shG3bp1, shSlu7, vector, HA‐G3bp1, or their different combinations were established via reinfection with the specified lentiviruses, followed by selection using the abovementioned concentration of puromycin and/or blasticidin.

### RNA Extraction and Quantitative Reverse Transcriptase Polymerase Chain Reaction (qRT–PCR)

Total RNA was extracted from cells using TRIzol reagent (Thermo Fisher Scientific) according to the manufacturer's instructions. cDNA was synthesized using HiScript II Q RT SuperMix for qPCR (Vazyme, Nanjing, China), according to the manufacturer's instructions. qRT–PCR was performed using SYBR Green Master Mix (Vazyme) via a LightCycler 480 real‐time PCR system (Roche Applied Science). All mRNA levels were normalized to those of GAPDH. The sequences of primers used for qRT–PCR are listed in Table S2 (Supporting Information).

### Western Blotting

Cells were lysed using RIPA buffer (1% NP‐40, 0.1% sodium dodecyl‐sulfate [SDS], 5 mm ethylenediaminetetraacetic acid [EDTA], 150 mm NaCl, and 50 mm Tris‐HCL; pH 8.0) supplemented with protease and phosphatase inhibitors (Sigma‐Aldrich, St. Louis, MO, USA). The lysates were cleared via centrifugation at 14 000 ×g and 4 °C for 15 min. The study used 8%–12% SDS–polyacrylamide gel electrophoresis (PAGE) to separate equal amounts of proteins, after which the separated proteins were transferred onto polyvinylidene difluoride membranes (Millipore, Bedford, MA, USA) and blocked with QuickBlock solution (Beyotime Biotechnology, Shanghai, China) for 10 min at room temperature (RT). The membranes were then incubated with primary antibodies, followed by incubation with horseradish peroxidase‐conjugated secondary antibodies. The signals were visualized using an enhanced chemiluminescence reagent (Tanon, Shanghai, China). Primary antibodies were used against G3BP1 (13057‐2‐AP, ProteinTech; 1:2000 dilution), MHC‐I (15240‐1‐AP, ProteinTech; 1:1000 dilution), SLU7 (12050‐1‐AP, ProteinTech; 1:500 dilution), Flag (14793, Cell Signaling Technology; 1:2000 dilution), HA (3724, Cell Signaling Technology; 1:2000 dilution), Akt (4691, Cell Signaling Technology; 1:500 dilution), p‐Akt^Thr308^ (9275, Cell Signaling Technology; 1:1000 dilution), p‐Akt^Ser473^ (4060, Cell Signaling Technology; 1:500 dilution), PIK3CA (4249, Cell Signaling Technology; 1:500 dilution), PIK3CB (3011, Cell Signaling Technology; 1:500 dilution), PIK3CD (34050, Cell Signaling Technology; 1:500 dilution), PIK3R1 (60225‐1‐Ig, ProteinTech; 1:500 dilution), GFP (2956, Cell Signaling Technology; 1:1000 dilution), eIF4G1 (15704‐1‐AP, ProteinTech; 1:500 dilution), PABPC1 (10970‐1‐AP, ProteinTech; 1:500 dilution), GAPDH (10494‐1‐AP, ProteinTech; 1:20 000 dilution), and alpha tubulin (11224‐1‐AP, ProteinTech; 1:5000 dilution).

### Flow Cytometry

The MHC‐I levels on the MB49 cell surface were detected using PE‐conjugated MHC class I (H‐2Kd/H‐2Dd) monoclonal antibody (#12‐5998‐82, Thermo Fisher Scientific), following the manufacturer's instructions. Briefly, MB49 cells were trypsinized and washed twice with PBS. Approximately half a million MB49 cells were resuspended in 100 µL of staining buffer (2% FBS in PBS) supplemented with 1.25 µL of MHC‐I antibody solution, and the cell suspension was incubated for 30 min at 4 °C under dark conditions. The cells were then washed twice and resuspended in 200 µL of PBS. CytoFLEX Flow Cytometer (Beckman Coulter, USA) were used for sample analysis and FlowJo 10.8.1 software for data analysis.

### In Vitro T Killing Assay

OT1CD8^+^ T cells were isolated from the spleen of OT1 mice (Cavens Biogle, Suzhou, China) using EasySepTM Mouse CD8+ T Cell Isolation Kit (#19853, STEMCELL Technologies), following the manufacturer's instructions. OT1CD8^+^ T cells were maintained in RPMI 1640 supplemented with 5% FBS, 55 µm β‐mercaptoethanol, 1% penicillin/streptomycin, and 10 ng mL^−1^ IL‐2. OT1CD8^+^ T cells were stimulated with 2 µg mL^−1^ SIINFEKL and 10 ng mL^−1^ IL‐2 for 3 days to generate CTLs. CTLs were cocultured with mCherry‐expressing MB49‐OVA cells for 24 h at a ratio of 2:1, with or without MK‐2206 (S1078, Selleck) or EGCG (S2250, Selleck). Cytotoxicity was assessed by calculating the percentage of 7‐AAD+ cells among mCherry+ cells using flow cytometry.

### Animal Experiments

Animal care and experiments were strictly performed according to the Guide for the Care and Use of Laboratory Animals and the Principles for the Utilization and Care of Vertebrate Animals. Five‐week‐old male C57BL/6J mice were purchased from Vital River Laboratories (Beijing, China) and housed them under specific pathogen‐free conditions (i.e., a 12/12 h light/dark cycle, 50% relative humidity, and temperature of 25–27 °C) with ad libitum access to food (standard chow) and water. The mice were acclimatized for 5 days before the experiment.

To determine the role of G3bp1 in tumor growth and CD8^+^ T cell infiltration, the mice were randomly divided into three groups of six mice each. MB49 cells with G3bp1 knockdown were trypsinized and washed twice with PBS. The mice were subcutaneously injected with 3 × 10^5^ MB49 cells (shNC or shG3bp1) suspended in 100 µL of PBS into the posterior flanks to establish the tumor‐bearing model. Tumor growth was monitored every 2 days, and tumor volume was calculated using the modified ellipsoidal formula (tumor volume = 1/2 [length × width^2^]). The mice were sacrificed on day 14, and the tumors were harvested, weighed, and imaged. The tumor of each mouse was fixed in 4% formaldehyde and embedded in paraffin for histological analysis.

To determine the effect of EGCG on the efficacy of anti‐PD‐1 antibody, 24 mice were further used to establish the above‐described tumor‐bearing model with wild‐type MB49 cells, and the mice were randomly categorized into four groups of six mice each. For treatment with EGCG and antibody, EGCG (50 mg kg^−1^) or vehicle was injected intraperitoneally on days 3, 5, 7, 9, 11, and 13 after inoculation with MB49 cells, and 200 µg of anti‐mouse PD‐1 antibody (BE0146, Bio X cell) or rat IgG (Bio X cell) was injected intraperitoneally on days 3, 6, 9, and 12. The body weight of each mouse was monitored on days 3, 9, and 13. The subsequent steps were performed as described above.

### RNA‐seq Analysis

RNA‐seq of BLCA cells with G3BP1 knockdown was performed by RiboBio (Guangzhou, China) using the Illumina NovaSeq 6000 System. Six gigabytes of clean data per sample were collected for RNA‐seq, and the clean reads were aligned with the human genome (hg19) using TopHat (version 2.0.13) software. The downstream signaling pathways of G3BP1 were revealed using KEGG pathway enrichment analysis and GSEA based on RNA‐seq data.

### RIP and RIP‐seq

RIP was performed using Magna RIP RNA‐Binding Protein Immunoprecipitation Kit (17‐700, Sigma‐Aldrich) according to the manufacturer's protocol. Briefly, UMUC3 cells with stable expression of HA‐G3BP1, SLU7‐Flag, or PABPC1‐GFP were lysed using RIP lysis buffer supplemented with a protease inhibitor cocktail and RNase inhibitor. Cell lysates were then incubated overnight at 4 °C under gentle rotation with antibodies against HA (26183, Thermo Fisher Scientific), Flag (8146, Cell Signaling Technology), GFP (66002‐1‐Ig, ProteinTech), or mouse IgG (B900620, ProteinTech). Each mixture was then incubated with protein A/G magnetic beads for 2 h at 4 °C under gentle rotation, and the immunoprecipitants were washed eight times with RIP wash buffer supplemented with a protease inhibitor cocktail and RNase inhibitor. Finally, total RNA was extracted using TRIzol reagent, after which RNA‐seq or qRT–PCR analysis were performed.

RIP‐seq was performed by RiboBio using the Illumina NovaSeq 6000 System. Ribosomal RNA sequences were removed. Twelve gigabytes of clean data per sample were obtained using RIP‐seq, and the clean reads were aligned to the human genome (hg19) using TopHat (version 2.0.13) software. Fisher's exact test was used to determine the statistical difference between HA‐G3BP1‐immunoprecipitated and input samples.

### Co‐IP, re‐IP, and MS Analysis

For co‐IP, cell lysates were prepared as described in section Western Blotting. Then, they were incubated overnight at 4 °C under gentle rotation with antibodies against Flag (8146, Cell Signaling Technology), HA (26183, Thermo Fisher Scientific), or mouse IgG (B900620, ProteinTech). Each mixture was then incubated with protein A/G magnetic beads (88803, Thermo Fisher Scientific) for 2 h at 4 °C under gentle rotation. Subsequently, the immunoprecipitants were washed eight times with RIPA buffer supplemented with protease inhibitors, after which they were analyzed using western blotting or MS analysis.

For re‐IP, UMUC3 cell lysates with simultaneous stable expression of SLU7‐Flag and HA‐G3BP1 were primarily immunoprecipitated with anti‐Flag antibody, after which they were blocked with 15 µg mL^−1^ Flag peptide (TP1273, TargetMol, USA) for 2 h at 4°C under gentle rotation. The resultant immunoprecipitants were subjected to re‐IP with anti‐HA antibody, washed as described previously, and analyzed via western blotting.

For MS analysis, the HA‐G3BP1 complex was obtained using co‐IP with anti‐HA antibody from UMUC3 cells stably expressing HA‐G3BP1. The purified immunoprecipitants were resolved via gradient SDS–PAGE, silver‐stained, and subjected to MS analysis. Briefly, the gels were minced and then destained with 50% acetonitrile in 50 mm ammonium bicarbonate. The proteins were reduced with 10 mm dithiothreitol at 56 °C and alkylated with 55 mm iodoacetamide at RT under dark conditions. Trypsin digestion was then performed for 20 h at 37 °C, after which the peptides were extracted with 1% trifluoroacetic acid in 50% acetonitrile. For MS analysis, the samples were vacuum‐dried and reconstituted with 0.1% formic acid. The pretreated samples were analyzed using nanoscale liquid chromatography coupled with tandem MS. Data were acquired using data‐dependent analysis and processed using PLGS software (Waters). The resultant peak list was searched against the National Center for Biotechnology Information database with the Mascot search engine.

### PLA

PLA was performed using Duolink In Situ Red Starter Kit (DUO92101, Sigma‐Aldrich) according to the manufacturer's instructions. Cells were seeded into confocal dishes and cultured overnight for adherence. They were then fixed with 4% paraformaldehyde for 15 min and penetrated with 0.5% Triton X‐100 for 10 min. The cells were blocked with a blocking solution for 1 h at 37 °C and coincubated overnight at 4 °C with antibodies against SLU7 (sc‐376985, Santa Cruz; 1:25 dilution) and G3BP1 (13057‐2‐AP, ProteinTech; 1:500 dilution). The cells were then incubated with PLA probes for 1 h at 37 °C, after which ligation was performed for 30 min at 37 °C. Amplification was performed using an amplification solution for 100 min at 37 °C. Finally, the samples were mounted using Fluoroshield with DAPI. Images were then captured using confocal laser scanning microscopy.

### Immunohistochemistry (IHC)

Formalin‐fixed and paraffin‐embedded BLCA tissues were sliced into 5‐µm thick sections and heated for 2 h at 60°C. The sections were deparaffinized with xylene and rehydrated in gradient ethanol, after which antigen retrieval was conducted in a pressure cooker at high pressure using EDTA buffer (pH 6.0–9.0). Subsequently, the sections were blocked with PBS supplemented with 3% bovine serum albumin (BSA) for 1 h at RT. The sections were then incubated with primary antibodies overnight at 4 °C, followed by incubation with secondary antibodies for 1 h at RT. Primary antibodies were used against SLU7 (NBP2‐42921, Novus Biologicals; 1:100 dilution) and G3BP1 (13057‐2‐AP, ProteinTech; 1:400 dilution). Further, the signals were visualized using DAB Visualization Kit (Dako Omnis, Santa Clara, CA, USA) according to the manufacturer's instructions. Finally, the sections were counterstained with hematoxylin. The H‐score was determined using HALO software (Indica Labs).

### IF Analysis and Immuno‐FISH

For IF analysis in cells, cell seeding, fixation, and penetration were performed similarly as described in section PLA. Subsequently, cells were blocked with 10% BSA for 1 h at RT and incubated with primary antibodies overnight at 4 °C. Then, the cells were washed and incubated with secondary antibodies for 1 h at RT, after which they were mounted using Fluoroshield with DAPI (F6057, Sigma‐Aldrich). For IF analysis in BLCA tissues, the sections were prepared and pretreated as described in section Immunohistochemistry (IHC); then, blocking, antibody incubation, and mounting were performed as described previously. Images were captured using confocal laser scanning microscopy. The following antibodies were used for IF analysis: G3BP1 (13057‐2‐AP, ProteinTech; 1:500 dilution), SLU7 (sc‐376985, Santa Cruz; 1:25 dilution), CD8 (GB11068, Servicebio; 1:200 dilution), Alexa Fluor 488‐conjugated anti‐rabbit IgG (A‐11034, ThermoFisher; 1:500 dilution), Alexa Fluor 555‐conjugated anti‐mouse IgG (A‐31570, ThermoFisher; 1:200 dilution), and Alexa Fluor 647‐conjugated anti‐rabbit IgG (A‐32733, ThermoFisher; 1:500 dilution).

For immuno‐FISH, IF analysis was performed as described previously. After incubation with secondary antibody, the cells were washed with PBS and subjected to single‐molecule inexpensive FISH, according to a previously reported method.^[^ ^36^ ^]^ Briefly, the cells were prehybridized with 15% deionized formamide in 1× saline sodium citrate (SSC) for 15 min at RT and incubated overnight at 37 °C with a hybridization solution. After hybridization, the samples were washed thrice with 15% formamide in 1× SSC at 37 °C for 30 min and mounted using Fluoroshield with DAPI. The sequences of the primary probes and FLAP probes are presented in Table S3 (Supporting Information).

### FISH–FRET

To identify the closed‐loop structure of a single mRNA, FISH–FRET method was established based on a previous report.^[^ ^37^ ^]^ Briefly, the cap and tail of an mRNA were labeled using Cy3 probes (donor) and Cy5 probes (receptor), respectively, and then evaluated its closed‐loop structure based on the FRET efficiency between the two probes. Theoretically, the FRET efficiency between Cy3 and Cy5 probes should decrease when the closed‐loop structure is disrupted.

Cell seeding, fixation, penetration, and blocking were performed similarly as described in section PLA. The Cy3 probes and Cy5 probes were created by annealing Cy3‐conjugated or Cy5‐conjugated FLAP probes with primary probes designed specifically for the cap or tail of an mRNA, following a previously reported method.^[^ ^36^ ^]^ Through FISH, the cap and tail of each mRNA were successively labeled with Cy3 and Cy5 probes, respectively. Briefly, cells were prehybridized with 15% deionized formamide in 1× SSC for 15 min at RT, after which they were incubated with a hybridization solution containing Cy3 probes, followed by incubation with Cy5 probes overnight at 37 °C. After hybridization, the samples were washed thrice with 15% formamide in 1× SSC at 37 °C for 30 min and mounted using Fluoroshield with DAPI. The sequences of the primary probes and FLAP probes are presented in Table S4 (Supporting Information).

FRET was performed using an inverted Zeiss LSM880 laser scanning confocal microscope with fast AiryScan (Zeiss, Germany) and the receptor photobleaching method. The cells used for FRET analysis were scanned, selected three random regions in the cytoplasmic region of a single cell, and continuously bleached the receptors (Cy5 probes) with a 633 laser at 100% intensity. The FRET efficiency in each region was calculated using ZEN 2.3 imaging software.

### Polysome Profiling Analysis

Polysome profiling analysis of the mRNAs of class IA PI3Ks was performed as previously described.^[^ ^38^ ^]^ Briefly, cells were incubated with 100 µg mL^−1^ cycloheximide (CHX) at 37 °C for 10 min. The cells were rinsed with ice‐cold PBS solution containing 100 µg mL^−1^ CHX and then lysed using 1 mL of lysis buffer (10 mM Tris‐HCl [pH 7.4], 5 mm MgCl_2_, 100 mm KCl, and 1% Triton X‐100) supplemented with 2 mm DTT, 100 µg mL^−1^ CHX, 50 U mL^−1^ RNase inhibitor, and EDTA‐free protease inhibitors, followed by centrifugation at 12 000 ×*g* and 4 °C for 10 min. The lysate was then applied to sucrose gradients of 10%–50% and ultracentrifuged at 4 °C and 36 000 rpm for 2 h using a Beckman SW41Ti rotor. Polysome profiles were measured via a UV monitor using Data Quest software at 254 nm with a piston gradient fractionator. RNA extraction and qRT–PCR were performed as previously described. The mRNA levels of class IA PI3Ks in each fraction were normalized to those of GAPDH. The squences of primers used for qRT–PCR are presented in Table S2 (Supporting Information).

### RNA Stability Assay

Cells were treated with 2 µg mL^−1^ actinomycin D (sbr00013, Sigma‐Aldrich). The cells were then harvested at 0, 4, 8, and 12 h after treatment with actinomycin D and performed RNA extraction and qRT–PCR analysis. The mRNA levels at different time points were determined by normalizing the mRNA expression to that detected at 0 h.

### Statistical Analysis

GraphPad Prism (Version 9.0) was used to construct graphs and perform statistical analyses. The mean values obtained for the control and experimental groups were analyzed for significant differences. All data were expressed as means ± standard deviation. The chi‐square test or Fisher's exact test was used to assess differences between variables. Statistical significance was determined using two‐tailed Student's *t*‐test, one‐way analysis of variance (ANOVA), or two‐way ANOVA. Survival analysis was performed using Kaplan–Meier analysis, Cox regression analysis, and log‐rank test. Correlations between variables were analyzed using Pearson's correlation test. A *P*‐value of <0.05 was considered to indicate statistical significance.

## Conflict of Interest

The authors declare no conflict of interest.

## Author Contributions

X.Z., J.C., and M.D. contributed equally to this work. X.Z. and J.C. designed the study, performed most of the experiments and drafted the manuscript; M.D., Zhenhua L., H.T., and Y.L. assisted the experiments and analyzed the data; K.N. and Y.P. performed the bioinformatic analysis; X.L. and Z.Z. provided clinical samples and relevant clinical information; T.K. and Zhuowei L. supervised the study and revised the manuscript. All authors have read and approved the final manuscript.

## Supporting information

Supporting Information

## Data Availability

The mass spectrometry proteomics data have been deposited to the ProteomeXchange Consortium via the PRIDE partner repository with the dataset identifier PXD035722. The raw sequence data have been deposited in the Genome Sequence Archive in the National Genomics Data Center China National Center for Bioinformation/Beijing Institute of Genomics, Chinese Academy of Sciences (HRA004476 to RNA‐seq data; HRA004475 to RIP‐seq data) that are publicly accessible at https://ngdc.cncb.ac.cn/gsa‐human. The datasets generated and/or analyzed during the current study are available from the corresponding author upon reasonable request.

## References

[1] L. M. C. Van Hoogstraten , A. Vrieling , A. G. Van Der Heijden , M. Kogevinas , A. Richters , L. A. Kiemeney , Nat. Rev. Clin. Oncol. 2023, 20, 287. 36914746 10.1038/s41571-023-00744-3

[2] M. Vlaming , L. A. L. M. Kiemeney , A. G. Van Der Heijden , Cancer Treat Res Commun 2020, 25, 100264. 33316558 10.1016/j.ctarc.2020.100264

[3] A. T. Lenis , P. M. Lec , K. Chamie , JAMA, J. Am. Med. Assoc. 2020, 324, 1980. 10.1001/jama.2020.17598 33201207

[4] A. M. Van Der Leun , D. S. Thommen , T. N. Schumacher , Nat. Rev. Cancer 2020, 20, 218. 32024970 10.1038/s41568-019-0235-4 PMC7115982

[5] N. Pishesha , T. J. Harmand , H. L. Ploegh , Nat. Rev. Immunol. 2022, 22, 751. 35418563 10.1038/s41577-022-00707-2

[6] Y. Bordon , Nat. Rev. Immunol. 2023, 23, 137. 36726035 10.1038/s41577-023-00842-4

[7] S. M. Toor , V. Sasidharan Nair , J. Decock , E. Elkord , Semin. Cancer Biol. 2020, 65, https://pubmed.ncbi.nlm.nih.gov/31265893/. 10.1016/j.semcancer.2019.06.021 31265893

[8] H. T.h. M. Timmers , L. Tora , Mol. Cell 2018, 72, 10. 30290147 10.1016/j.molcel.2018.08.023

[9] a) R. G. H. Lindeboom , M. Vermeulen , B. Lehner , F. Supek , Nat. Genet. 2019, 51, 1645; 31659324 10.1038/s41588-019-0517-5 PMC6858879

[10] a) L. A. Passmore , J. Coller , Nat. Rev. Mol. Cell Biol. 2022, 23, 93; 34594027 10.1038/s41580-021-00417-y PMC7614307

[11] Y. Zhao , C. Mir , Y. Garcia‐Mayea , R. Paciucci , H. Kondoh , M. E. Lleonart , Semin. Cancer Biol. 2022, 86, 431. 35124196 10.1016/j.semcancer.2022.01.010

[12] A. Necchi , R. W. Joseph , Y. Loriot , J. Hoffman‐Censits , J. L. Perez‐Gracia , D. P. Petrylak , C. L. Derleth , D. Tayama , Q. Zhu , B. Ding , C. Kaiser , J. E. Rosenberg , Ann. Oncol. 2017, 28, 3044. 28950298 10.1093/annonc/mdx518 PMC5834063

[13] J. Guillén‐Boixet , A. Kopach , A. S. Holehouse , S. Wittmann , M. Jahnel , R. Schlüßler , K. Kim , I. R. E. A. Trussina , J. Wang , D. Mateju , I. Poser , S. Maharana , M. Ruer‐Gruß , D. Richter , X. Zhang , Y.‐T. Chang , J. Guck , A. Honigmann , J. Mahamid , A. A. Hyman , R. V. Pappu , S. Alberti , T. M. Franzmann , Cell 2020, 181, 346. 32302572 10.1016/j.cell.2020.03.049 PMC7181197

[14] a) X. Zhang , C. Yan , J. Hang , L. I. Finci , J. Lei , Y. Shi , Cell 2017, 169, 918; 28502770 10.1016/j.cell.2017.04.033

[15] a) Y. Wang , X. Wang , X. Cui , Y. Zhuo , H. Li , C. Ha , L. Xin , Y. Ren , W. Zhang , X. Sun , L. Ge , X. Liu , J. He , T. Zhang , K. Zhang , Z. Yao , X.i Yang , J. Yang , Sci. Adv. 2020, 6, aa5412; 10.1126/sciadv.aba5412 PMC7259962 32917674

[16] S. Santasusagna , S. Zhu , V. Jawalagatti , M. Carceles‐Cordon , A. Ertel , S. Garcia‐Longarte , W.‐M. Song , N. Fujiwara , P. Li , I. Mendizabal , D. P. Petrylak , W. K. Kelly , E. P. Reddy , L. Wang , M. J. Schiewer , A. Lujambio , J. Karnes , K. E. Knudsen , C. Cordon‐Cardo , H. Dong , H. Huang , A. Carracedo , Y. Hoshida , V. Rodriguez‐Bravo , J. Domingo‐Domenech , Cancer Discov 2023, OF1, 10.1158/2159-8290.CD-23-0306. PMC10714140 37676710

[17] J. Zhang , G.e Zhang , W. Zhang , L.u Bai , L. Wang , T. Li , L.i Yan , Y. Xu , D. Chen , W. Gao , C. Gao , C. Chen , M. Ren , Y. Jiao , H. Qin , Y.u Sun , L. Zhi , Y. Qi , J. Zhao , Q. Liu , H. Liu , Y. Wang , Cell Death Differ. 2022, 29, 2247. 35538152 10.1038/s41418-022-01012-0 PMC9613699

[18] Y. Sun , Z. Li , W. Wang , X. Zhang , W. Li , G. Du , J. Yin , W. Xiao , H. Yang , Front Immunol 2022, 13, 957865. 36059530 10.3389/fimmu.2022.957865 PMC9433931

[19] J. Luzha , N. Nass , P. Czapiewski , N. Schroeder , T. Kalinski , M. Schostak , C. Schatz , B. Jandrig , J. Haybaeck , Anticancer Res. 2023, 43, 1437. 36974813 10.21873/anticanres.16292

[20] M. Cheng , L.u Sheng , Q. Gao , Q. Xiong , H. Zhang , M. Wu , Y.u Liang , F. Zhu , Y. Zhang , X. Zhang , Q. Yuan , Y. Li , Oncogene 2019, 38, 3667. 30659266 10.1038/s41388-019-0683-z

[21] a) N. Sivaram , P. A. Mclaughlin , H. V. Han , O. Petrenko , Y.a‐P. Jiang , L. M. Ballou , K. Pham , C. Liu , A. W. M. Van Der Velden , R. Z. Lin , J. Clin. Invest. 2019, 129, 3264; 31112530 10.1172/JCI123540 PMC6668699

[22] a) J. W. Fischer , V. F. Busa , Y. Shao , A. K. L. Leung , Mol. Cell 2020, 78, 70; 32017897 10.1016/j.molcel.2020.01.021 PMC8055448

[23] a) N. Amrani , S. Ghosh , D. A. Mangus , A. Jacobson , Nature 2008, 453, 1276; 18496529 10.1038/nature06974 PMC2587346

[24] J. Choe , S. Lin , W. Zhang , Q.i Liu , L. Wang , J. Ramirez‐Moya , P. Du , W. Kim , S. Tang , P. Sliz , P. Santisteban , R. E. George , W. G. Richards , K.‐K. Wong , N. Locker , F. J. Slack , R. I. Gregory , Nature 2018, 561, 556. 30232453 10.1038/s41586-018-0538-8 PMC6234840

[25] a) J.‐H. Shim , Z.‐Y. Su , J.‐I. Chae , D. J. Kim , F. Zhu , W.‐Y.a Ma , A. M. Bode , C. S. Yang , Z. Dong , Cancer Prev. Res. 2010, 3, 670; 10.1158/1940-6207.CAPR-09-0185 20424128

[26] a) J. Mclarty , R. L. H. Bigelow , M. Smith , D. Elmajian , M. Ankem , J. A. Cardelli , Cancer Prev. Res. 2009, 2, 673; 10.1158/1940-6207.CAPR-08-0167 19542190

[27] a) L. C. Reineke , R. E. Lloyd , J. Virol. 2015, 89, 2575; 25520508 10.1128/JVI.02791-14 PMC4325707

[28] H. Cai , X. Liu , F. Zhang , Q.‐Y. Han , Z.‐S. Liu , W. Xue , Z.‐L. Guo , J.‐M. Zhao , L.‐M. Sun , N.a Wang , J. Mao , K. He , T. Xia , Y. Chen , L. Chen , A.i‐L. Li , T. Zhou , X.‐M. Zhang , W.‐H. Li , T. Li , J. Immunol. 2021, 206, 2453. 33941659 10.4049/jimmunol.2001111

[29] a) D. A. Fruman , H. Chiu , B. D. Hopkins , S. Bagrodia , L. C. Cantley , R. T. Abraham , Cell 2017, 170, 605; 28802037 10.1016/j.cell.2017.07.029 PMC5726441

[30] a) I. Vivanco , C. L. Sawyers , Nat. Rev. Cancer 2002, 2, 489; 12094235 10.1038/nrc839

[31] X. He , J. Yuan , Y. Wang , Nucleic Acids Res. 2021, 49, 11323. 34614161 10.1093/nar/gkab873 PMC8565330

[32] N. Shomron , M. Alberstein , M. Reznik , G. Ast , J. Cell Sci. 2005, 118, 1151. 15728250 10.1242/jcs.01720

[33] M. Gárate‐Rascón , M. Recalde , M. Jimenez , M. Elizalde , M. Azkona , I. Uriarte , M. U. Latasa , R. Urtasun , I. Bilbao , B. Sangro , C. Garcia‐Ruiz , J. C. Fernandez‐Checa , F. J. Corrales , A. Esquivel , A. Pineda‐Lucena , M. G. Fernández‐Barrena , M. A. Ávila , M. Arechederra , C. Berasain , Hepatology 2021, 74, 2791. 34170569 10.1002/hep.32029

[34] S. S. Gu , W. Zhang , X. Wang , P. Jiang , N. Traugh , Z. Li , C. Meyer , B. Stewig , Y. Xie , X. Bu , M. P. Manos , A. Font‐Tello , E. Gjini , A. Lako , K. Lim , J. Conway , A. K. Tewari , Z. Zeng , A. D. Sahu , C. Tokheim , J. L. Weirather , J. Fu , Y. Zhang , B. Kroger , J. H. Liang , P. Cejas , G. J. Freeman , S. Rodig , H. W. Long , B. E. Gewurz , et al., Cancer Discov 2021, 11, 1524. 33589424 10.1158/2159-8290.CD-20-0812 PMC8543117

[35] K. Nagaraju , N. Raben , L. Loeffler , T. Parker , P. J. Rochon , E. Lee , C. Danning , R. Wada , C. Thompson , G. Bahtiyar , J. Craft , R. Hooft Van Huijsduijnen , P. Plotz , Proc. Natl. Acad. Sci. USA 2000, 97, 9209. 10922072 10.1073/pnas.97.16.9209 PMC16847

[36] N. Tsanov , A. Samacoits , R. Chouaib , A.‐M. Traboulsi , T. Gostan , C. Weber , C. Zimmer , K. Zibara , T. Walter , M. Peter , E. Bertrand , F. Mueller , Nucleic Acids Res. 2016, 44, e165. 27599845 10.1093/nar/gkw784 PMC5159540

[37] a) G. Wadsworth , R. Parikh , H. Kim , Bio Protoc 2018, 8, e2867; 10.21769/BioProtoc.2867 PMC8275227 34285981

[38] C. Han , L. Sun , Q. Pan , Y. Sun , W. Wang , Y. Chen , STAR Protoc 2022, 3, 101037. 34977682 10.1016/j.xpro.2021.101037 PMC8683657

