# Supplementary material for: G3BP1 and SLU7 Jointly Promote Immune Evasion by Downregulating MHC‐I via PI3K/Akt Activation in Bladder Cancer
Source: Adv Sci (Weinh). 2023 Dec 12;11(7):2305922. doi: 10.1002/advs.202305922 (PMC10870071; doi:10.1002/advs.202305922)
Supplement: Supplementary file 1 — Supporting Information [file ADVS-11-2305922-s001.pdf]

ADVANCED SCIENCE
Open Access

# Supporting Information

for *Adv. Sci.*, DOI 10.1002/advs.202305922

G3BP1 and SLU7 Jointly Promote Immune Evasion by Downregulating MHC-I via PI3K/Akt Activation in Bladder Cancer

*Xianchong Zheng, Jiawei Chen, Minhua Deng, Kang Ning, Yulu Peng, Zhenhua Liu, Xiangdong Li, Zhaohui Zhou, Huancheng Tang, Yaoying Li, Tiebang Kang* and Zhuowei Liu**

**Supplementary Figures**

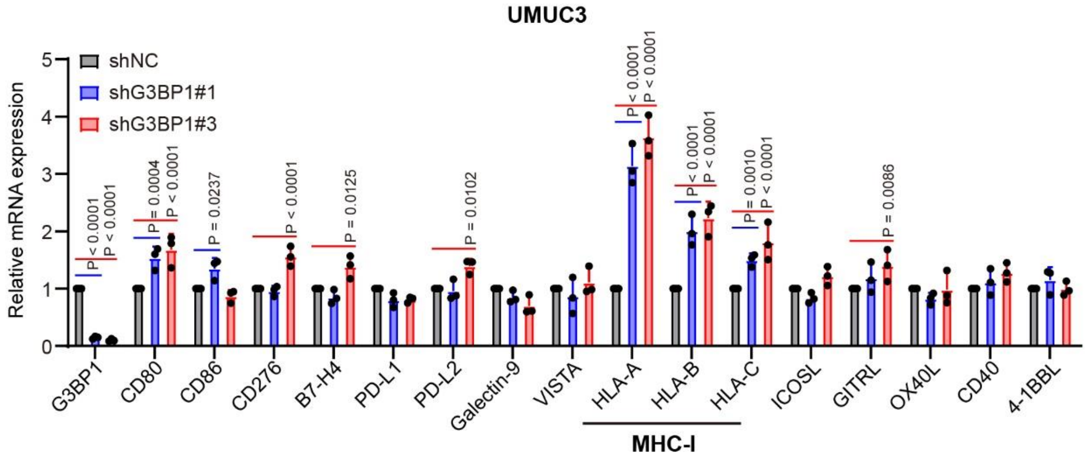


**Figure S1. G3BP1 Knockdown in UMUC3 cells upregulates the mRNA levels of MHC-I.** UMUC3 cells with G3BP1 knockdown were generated by two independent shRNAs that introduced by lentiviral infection. The mRNA levels of G3BP1 and immune checkpoints in UMUC3 cells with or without G3BP1 knockdown were evaluated using qRT–PCR analysis. The relative mRNA expression was normalized based on that of GAPDH. Error bars represent SD (n = 3). *P*-values are presented and were calculated using two-way ANOVA.

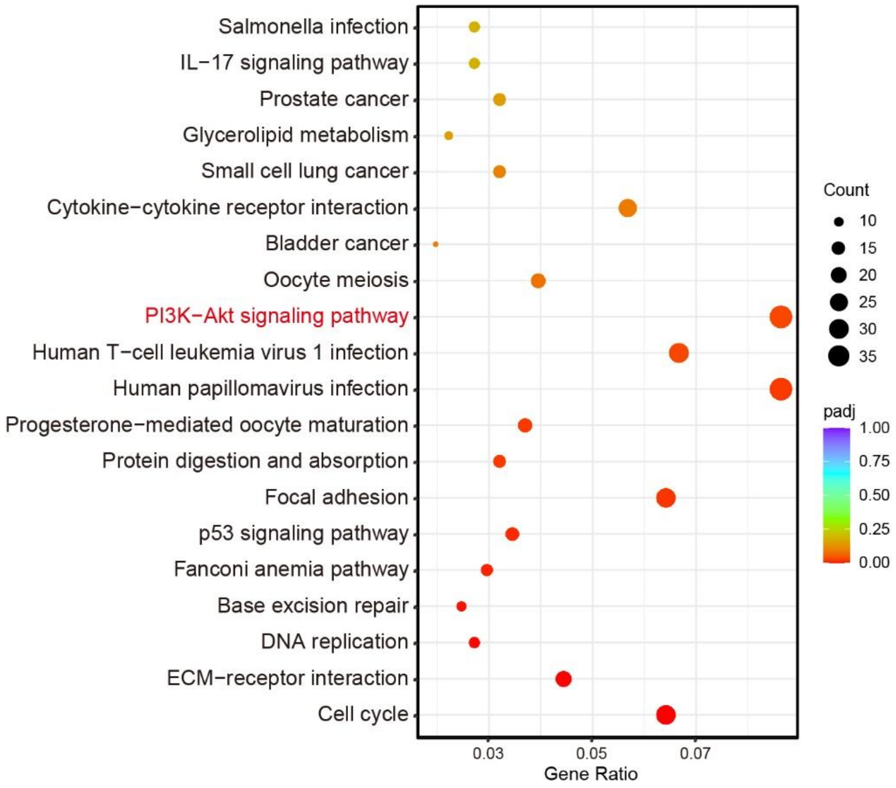


**Figure S2. G3BP1 knockdown inhibits the PI3K/Akt signaling pathway.** UMUC3 cells with G3BP1 knockdown were subjected to RNA-seq analysis. The downstream signaling pathways of G3BP1 were analyzed via KEGG pathway enrichment analysis.

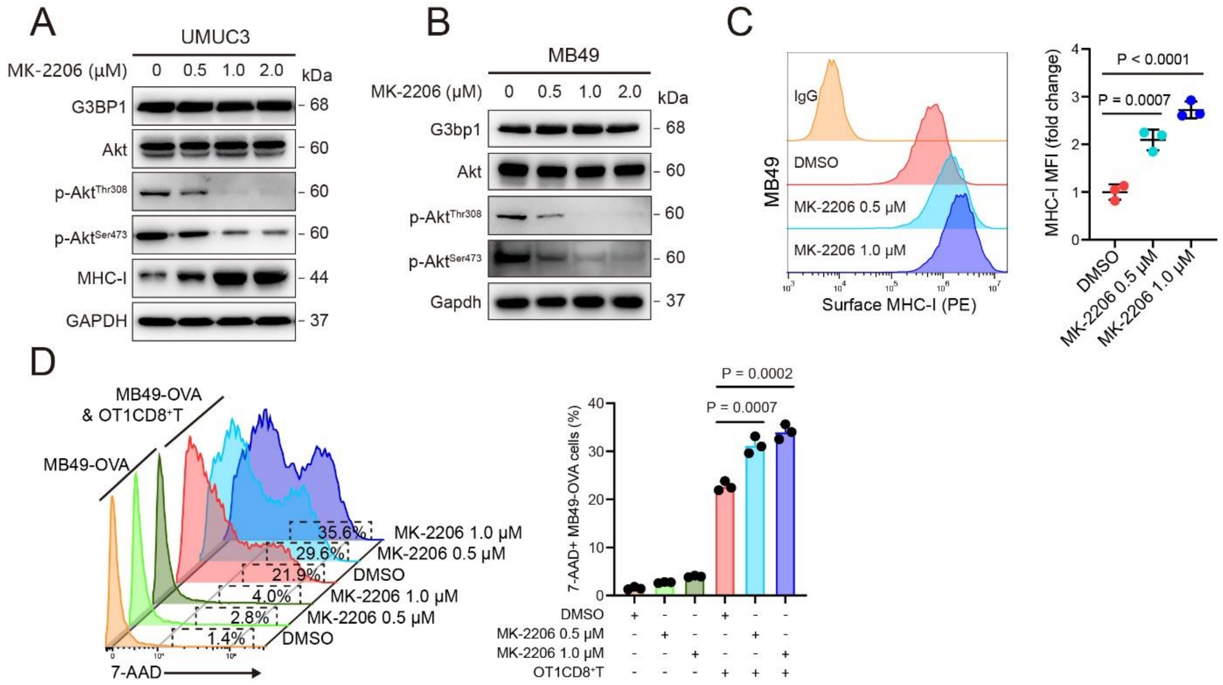


**Figure S3. Akt inhibition by MK-2206 upregulates MHC-I and impedes immune evasion. A and B**) UMUC3 cells and MB49 cells were treated with MK-2206 with the indicated concentrations for 24 h, the expression levels of G3BP1, SLU7, total Akt, p-Akt$^{Thr308}$, p-Akt$^{Ser473}$, and/or MHC-I were detected using western blotting. GAPDH was used as the internal control. **C**) The expression of MHC-I on the surface of MB49 cells in (B) was analyzed using flow cytometry. MHC-I expression was quantified based on the mean fluorescence intensity (MFI). **D**) mCherry-expressing MB49-OVA cells with MK-2206 treatment were co-cultured with or without antigen-activated CTLs for 24 h. Cytotoxicity was determined by analyzing the percentage of 7-AAD+ cells among mCherry+ cells. Error bars represent SD (n = 3 in C and D). *P*-values are presented and were calculated using one-way ANOVA (C and D).

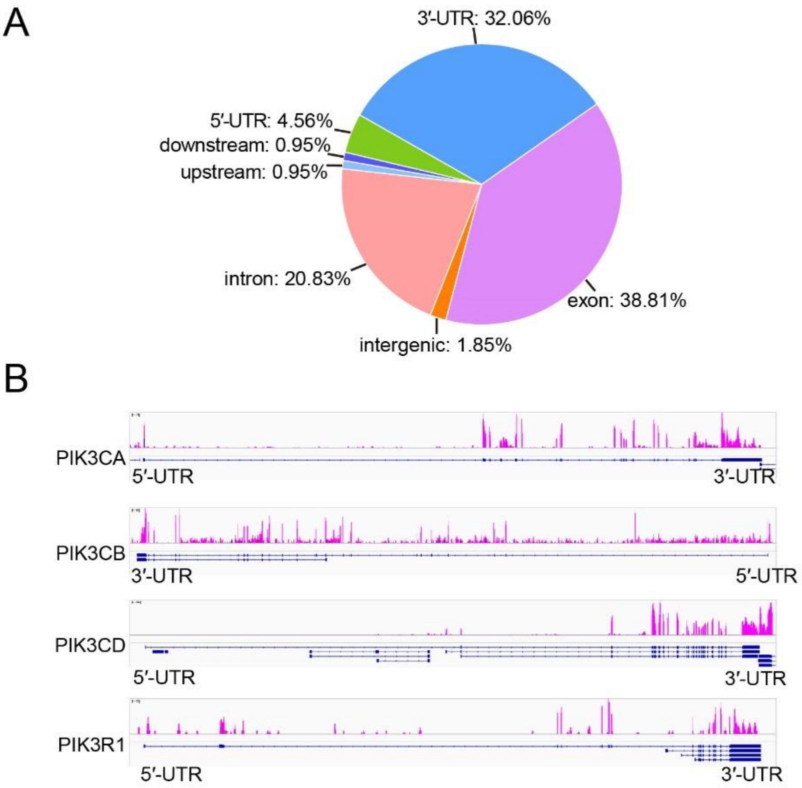


**Figure S4. G3BP1 binds to the mRNAs of class IA PI3Ks via multiple binding peaks. A**) Pie chart showing the annotation results of the binding peaks of G3BP1 on potential RNAs. **B**) The binding peaks of G3BP1 on the mRNAs of class IA PI3Ks were visualized using Integrative Genomics Viewer (https://software.broadinstitute.org/software/igv/).

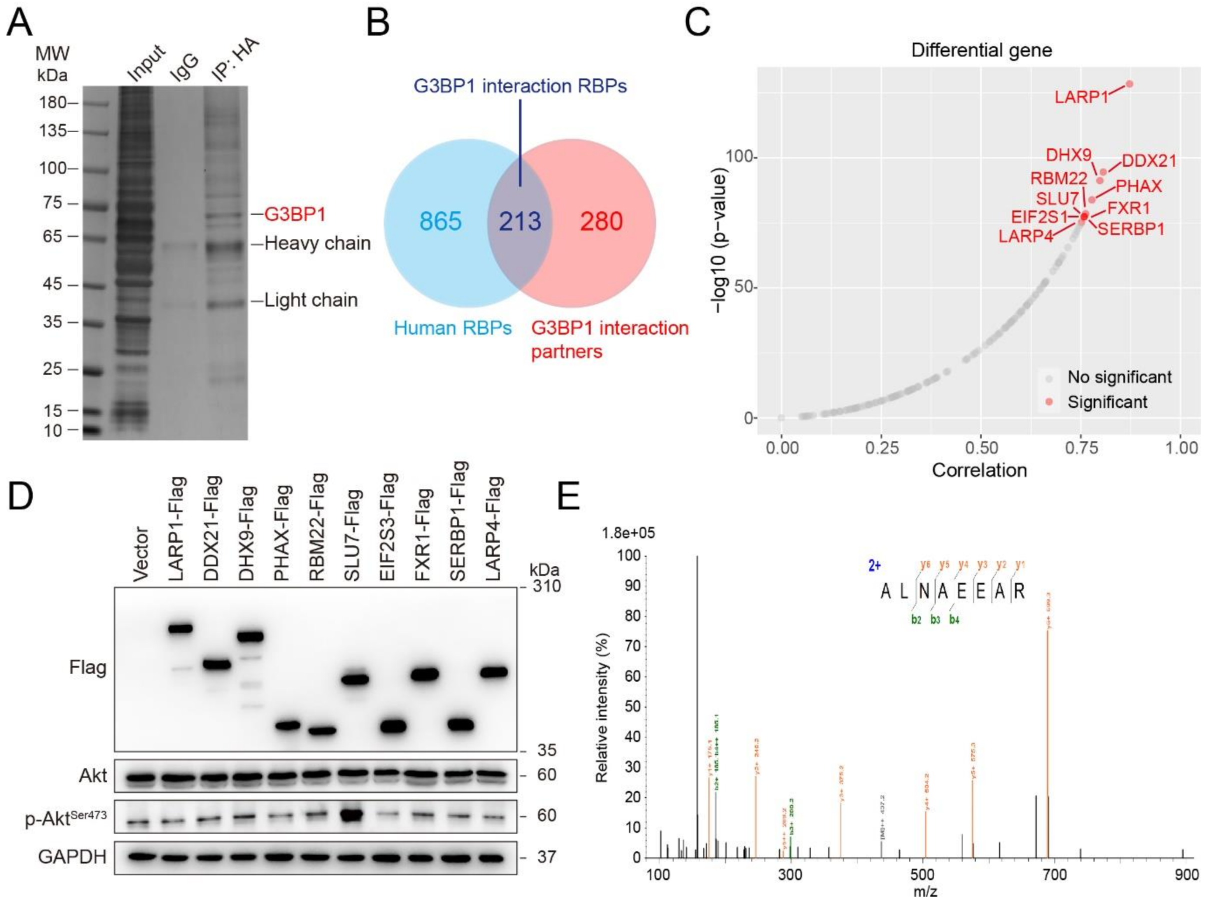


**Figure S5. SLU7 interacts with G3BP1 and promotes Akt activation. A**) Cell lysates from UMUC3 cells with HA-G3BP1 stable expression were immunoprecipitated with anti-HA antibodies, and the immunoprecipitants were subjected to SDS-PAGE and silver-stained. The differential protein bands were retrieved and identified using MS. **B**) The Venn diagram illustrates the overlap between human RBPs and the 493 proteins identified by MS. Since G3BP1 localizes exclusively in the cytoplasm, we have excluded RBPs that do not localize in the cytoplasm. **C**) The correlation between the expression of G3BP1 and G3BP1-interacting RBPs in BLCA was analyzed based on TCGA dataset. The RBPs among the top hits in terms of positive correlation with G3BP1 were displayed. **D**) Plasmids expressing Flag-tagged LARP1, DDX21, DHX9, PHAX, RBM22, SLU7, EIF2S3, FXR1, SERBP1 and LARP4 were transfected to UMUC3 cells, and the expression of Flag, Akt, p-Akt$^{Ser473}$ was detected by western blotting. GAPDH was used as the internal control. **E**) A peptide segment diagram of SLU7 identified by MS.

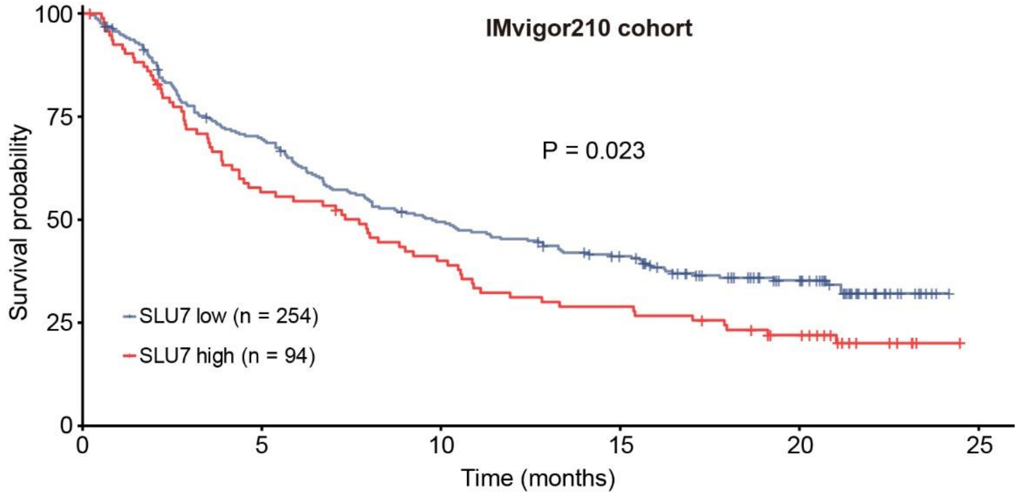


**Figure S6. High SLU7 expression is linked to unfavorable outcomes in patients from the IMvigor210 cohort.** The impact of SLU7 on the survival of BLCA patients receiving anti-PD-L1 antibody therapy was assessed through Kaplan-Meier analysis using the best cutoff. The mRNA expression data were obtained from the IMvigor210 cohort, and the resulting Kaplan-Meier curve for SLU7 in this cohort is presented.

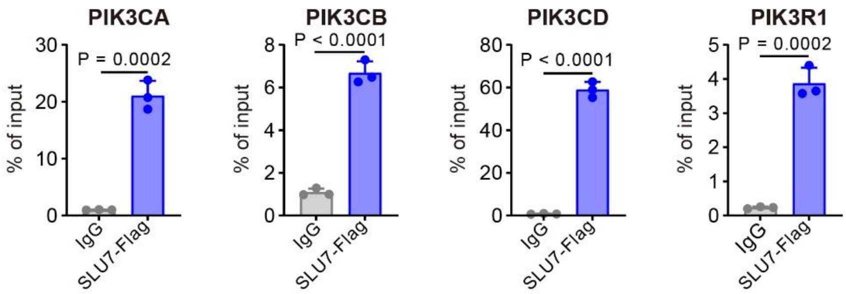


**Figure S7. SLU7 binds to the mRNAs of class IA PI3Ks.** A RIP assay was performed in UMUC3 cells with SLU7-Flag stable expression with anti-Flag antibodies. The mRNA levels of class IA PI3Ks in the immunoprecipitants were evaluated using qRT–PCR analysis. Error bars represent SD (n = 3). *P*-values are presented and were calculated using unpaired, two-tailed Student’s *t*-test.

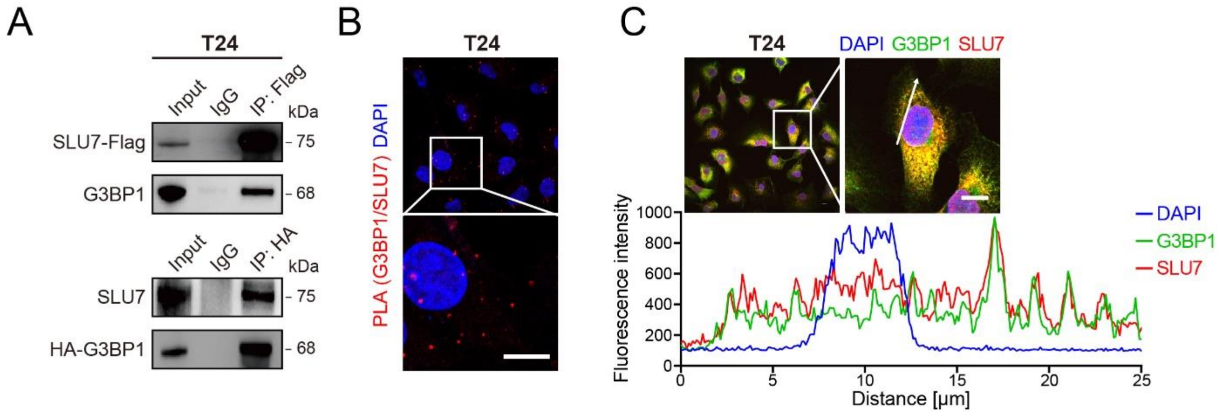


**Figure S8. SLU7 interacts with G3BP1 in T24 cells. A**) Cell lysates from T24 cells with stable expression of SLU7-Flag or HA-G3BP1 were immunoprecipitated with anti-Flag or anti-HA antibodies and immunoblotting was conducted with the indicated antibodies. **B**) The interaction of G3BP1 and SLU7 in T24 cells was evaluated using PLA. **C**) The locations of G3BP1 and SLU7 in T24 was analyzed using IF analysis. Scale bar, 10 μm. The Fluorescence intensity curves of DAPI (blue), G3BP1 (green), and SLU7 (red) that crossed the arrow were analyzed.

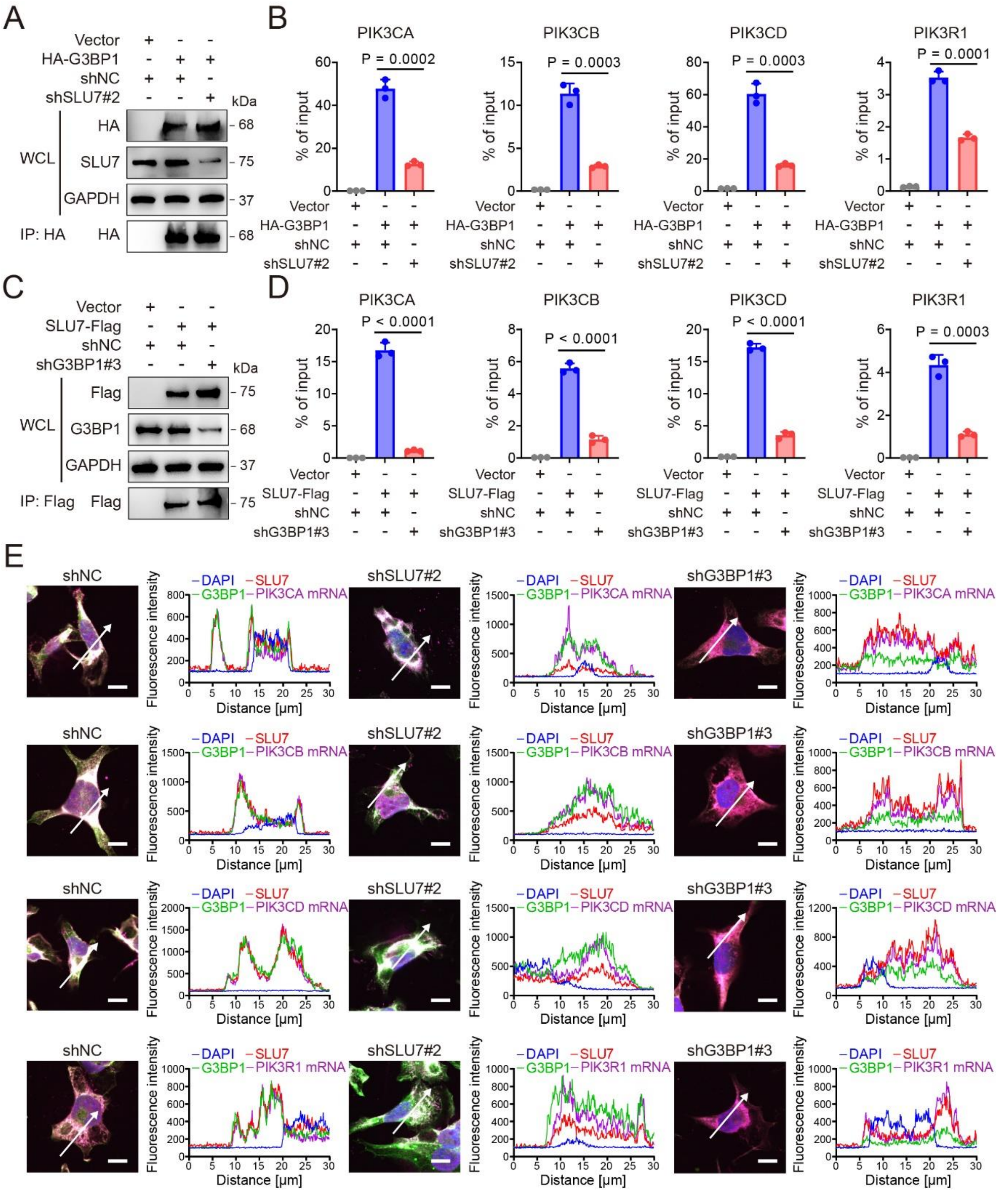


**Figure S9. G3BP1 and SLU7 interdependently bind to the mRNAs of class IA PI3Ks. A**) Cell lysates from HA-G3BP1 stable expression UMUC3 cells with or without SLU7 knockdown were immunoprecipitated with anti-HA antibody. The expression levels of HA and SLU7 in whole cell lysates and immunoprecipitants were detected using western blotting. **B**) The mRNA levels of class IA PI3Ks in the immunoprecipitants were evaluated using qRT–PCR analysis. **C**) Cell lysates from SLU7-Flag stable expression UMUC3 cells with or without G3BP1 knockdown were

immunoprecipitated with anti-Flag antibody. The expression levels of Flag and G3BP1 in whole cell lysates and immunoprecipitants were detected using western blotting. **D**) The mRNA levels of class IA PI3Ks in the immunoprecipitants were evaluated using qRT–PCR analysis. **E**) The locations of G3BP1, SLU7, and the mRNA of PI3Ks in UMUC3 cells with SLU7 knockdown or G3BP1 knockdown was analyzed using immuno-FISH. Scale bar, 10 μm. The fluorescence intensity curves of DAPI (blue), G3BP1 (green), SLU7 (red), and mRNA (purple) that crossed the arrows were analyzed. Error bars represent SD (n = 3 in B and D). *P*-values are presented and were calculated using unpaired, two-tailed Student's *t*-test (B and D).

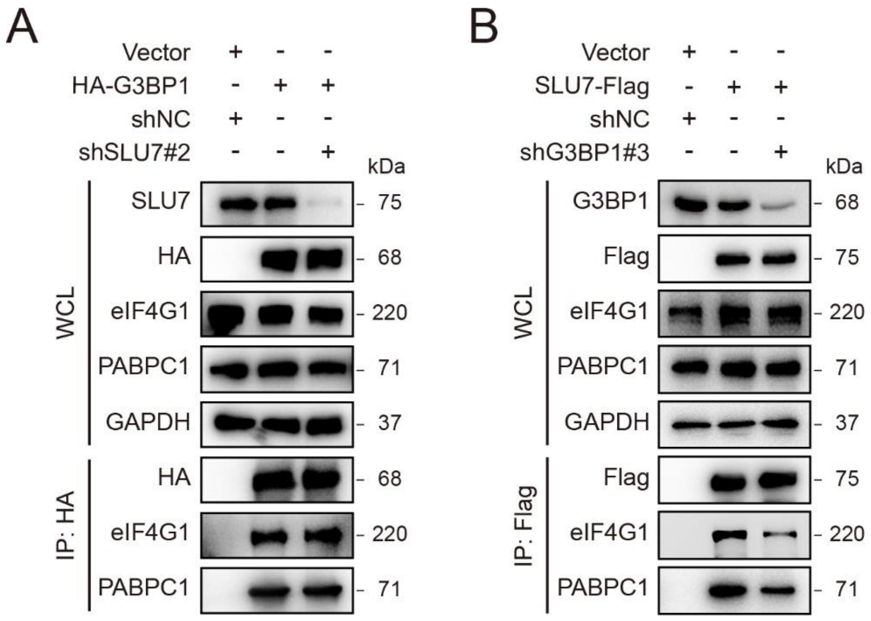


**Figure S10. The binding of SLU7 with PABPC1/eIF4G1 depends on G3BP1. A**) Cell lysates from HA-G3BP1 stable expression UMUC3 cells with or without SLU7 knockdown were immunoprecipitated with anti-HA antibodies and immunoblotted with the indicated antibodies. **B**) Cell lysates from SLU7-Flag stable expression UMUC3 cells with or without G3BP1 knockdown were immunoprecipitated with anti-Flag antibodies and immunoblotted with the indicated antibodies.

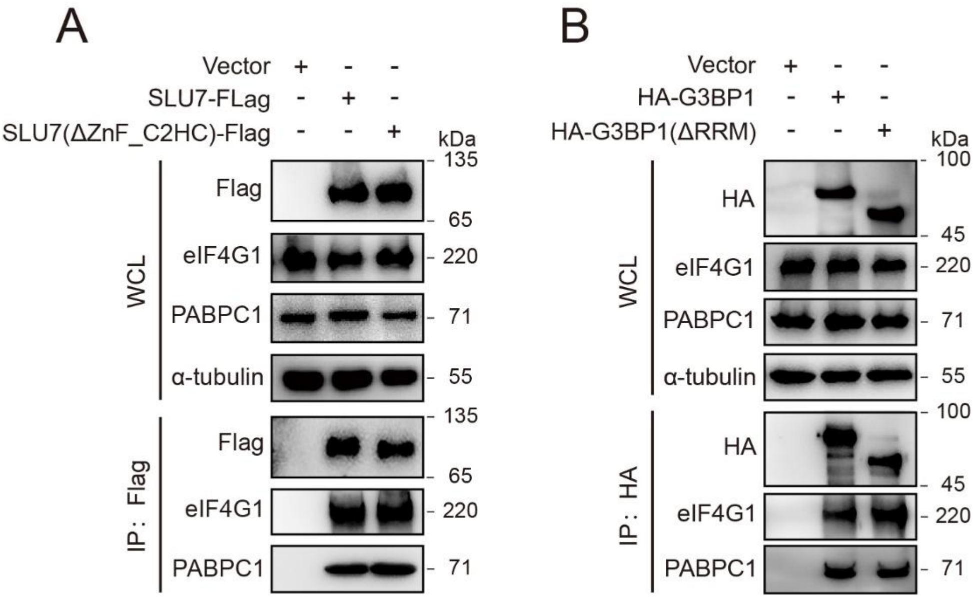


**Figure S11. The binding of G3BP1-SLU7 to PABPC1/eIF4G1 does not depend on the RBDs of G3BP1 and SLU7. A**) Cell lysates from HEK293T cells stably expressing SLU7-Flag or SLU7 (ΔZnF-C2HC)-Flag were immunoprecipitated with anti-Flag antibodies and immunoblotted with the indicated antibodies. **B**) Cell lysates from HEK293T cells stably expressing HA-G3BP1 or HA-G3BP1 (ΔRRM) were immunoprecipitated with anti-HA antibodies and immunoblotted with the indicated antibodies.

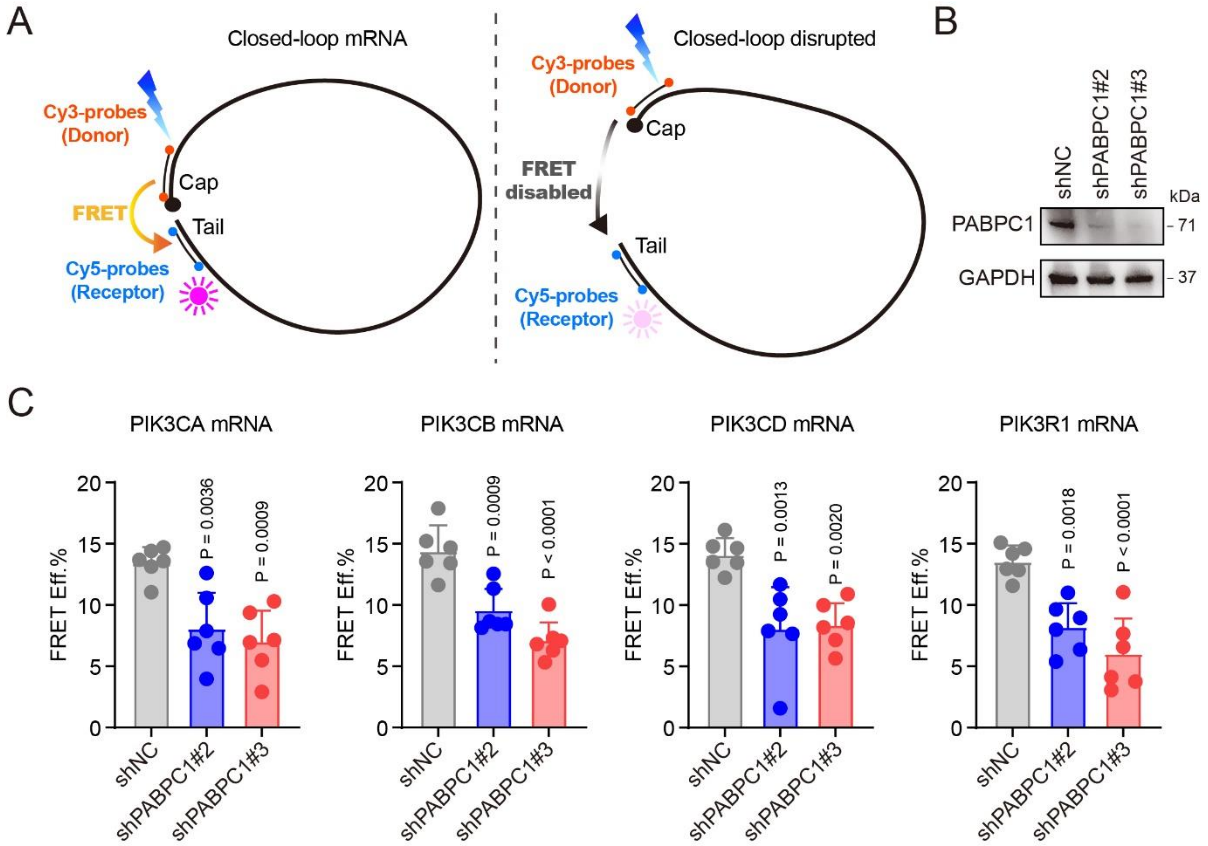


**Figure S12. PABPC1 knockdown destroys the closed-loop structure of class IA PI3K mRNAs. A**) A graphical model showing the basic principle of the FISH–FRET method for identifying the closed-loop structure of specific mRNAs. **B**) The expression of PABPC1 in PABPC1-knockdown UMUC3 cells was detected using western blotting. **C**) The FRET efficiency between Cy3-probes and Cy5-probes on the class IA PI3Ks mRNA in UMUC3 cells with PABPC1 knockdown was analyzed. Error bars represent SD (n = 6). *P*-values are presented and were calculated using one-way ANOVA (C).

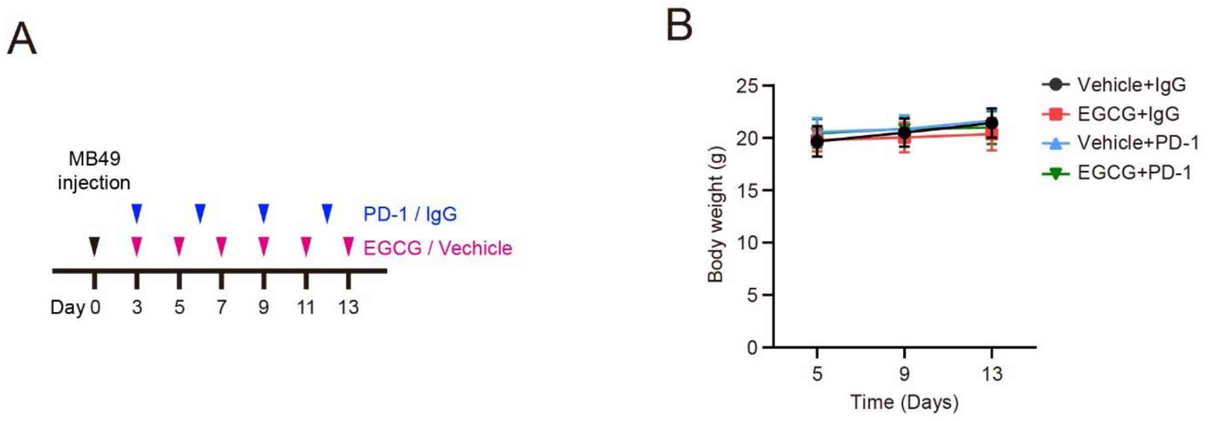


**Figure S13. Treatment with EGCG and/or anti-PD-1 antibody did not affect the weight of the mice. A**) Study design of C57BL/6J mice bearing subcutaneous bladder tumors (implanted with $3 \times 10^5$ MB49 cells) received EGCG, vehicle, IgG, or anti-PD-1 antibody therapy. **B**) The body weight of the mice on the indicated days. Error bars represent SD (n = 6).

## Supplementary Tables

### Table S1. The target sequences of shRNAs.

| shRNA ID | Targeting sequence | Species |
|---|---|---|
| shG3BP1#1 | TTAGTCTTTCACTTCCAATTT | human |
| shG3BP1#3 | AGTGCGAGAACAACGAATAAA | human |
| shSLU7#2 | CACAGGAGATACCATTTCAAT | human |
| shSLU7#4 | CAGAAGTTGATGAAGAAGGAA | human |
| shPABPC1#2 | AGCTGTTCCCAACCCTGTAAT | human |
| shPABPC1#3 | CCGCACCGTTCCACAGTATAA | human |
| shG3bp1#2 | CCTCAGAGAGATCAGAGAGTT | mouse |
| shG3bp1#4 | TCTCGGAATAGTCTATTATAA | mouse |
| shSlu7#1 | CCCTCATATTCCTCAGTATAT | mouse |
| shSlu7#2 | CCCTACAACAGCATCTATGAA | mouse |
| non-targeting shRNA (shNC) | CAACAAGATGAAGAGCACCAA | N.A. |

### Table S2. The sequences of primers used for qRT–PCR.

| Gene ID | Forward primer sequence (5′-3′) | Reverse primer sequence (5′-3′) |
|---|---|---|
| CD80 | AAACTCGCATCTACTGGCAAA | GGTTCTTGTACTCGGGCCATA |
| CD86 | CCATCAGCTTGTCTGTTTCATTCC | GCTGTAATCCAAGGAATGTGGTC |
| CD276 | CTGGCTTTCGTGTGCTGGAGAA | GCTGTCAGAGTGTTTCAGAGGC |
| B7-H4 | CTCACAGATGCTGGCACCTACA | GCAAGGTCTCTGAGCTGGCATT |
| PD-L1 | TGCCGACTACAAGCGAATTACTG | CTGCTTGTCCAGATGACTTCGG |
| PD-L2 | CTCGTTCCACATACCTCAAGTCC | CTGGAACCTTTAGGATGTGAGTG |
| Galectin-9 | ACACCCAGATCGACAACTCCTG | CAAACAGGTGCTGACCATCCAC |
| VISTA | AGATGCACCATCCAACTGTGTGG | AGGCAGAGGATTCCTACGATGC |
| HLA-A | AGATACACCTGCCATGTGCAGC | GATCACAGCTCCAAGGAGAACC |
| HLA-B | CTGCTGTGATGTGTAGGAGGAAG | GCTGTGAGAGACACATCAGAGC |

| | | |
|---|---|---|
| HLA-C | GGAGACACAGAAGTACAAGCGC | ACATCCTCTGGAGGGTGTGAGA |
| ICOSL | GTTTCACTGCCTGGTGTTGAGC | ACGACGGGCACGCTGAAGTTTG |
| GITRL | AGTGGCTCCCAATGCAAACTA | TATACAGCCGCACCTCAAAAG |
| OX40L | GGTCAGGTCTGTCAACTCCTT | CATCCAGGGAGGTATTGTCAGT |
| CD40 | TTGGGGTCAAGCAGATTGCTA | GCAGATGACACATTGGAGAAGA |
| 4-1BBL | GGCTGGAGTCTACTATGTCTTCT | ACCTCGGTGAAGGGAGTCC |
| G3BP1 | CGGGCGGGAATTTGTGAGA | TCTGTCCGTAGACTGCATCTG |
| SLU7 | AGTTAATGCTGCACCCCTATCG | GCCCAATTTTCGCTGTTCTTCTA |
| PIK3CA | CCACGACCATCATCAGGTGAA | CCTCACGGAGGCATTCTAAAGT |
| PIK3CB | TATTTGGACTTTGCGACAAGACT | TCGAACGTACTGGTCTGGATAG |
| PIK3CD | AAGGAGGAGAATCAGAGCGTT | GAAGAGCGGCTCATACTGGG |
| PIK3R1 | AAGAAGTTGAACGAGTGGTTGG | GCCCTGTTTACTGCTCTCCC |
| GAPDH | GGAGCGAGATCCCTCCAAAAT | GGCTGTTGTCATACTTCTCATGG |

**Table S3. The sequences of the primary probes and FLAP probes used for smiFISH.**

| Probe ID | Sequence (5′-3′) |
|---|---|
| Cy5-tagged FLAP | Cy5-AATGCATGTCGACGAGGTCCGAGTGTAA-Cy5 |
| PIK3CA#1 | TGTCTGGGTTCTCCCAATTCAACCACAGTTACACTCGGACCTCGTC GACATGCATT |
| PIK3CA#2 | GCCTTTTTGCAGAGGACATAATTCGACACTCTTACACTCGGACCTC GTCGACATGCATT |
| PIK3CA#3 | CAAGCCTGAGGTTTCCTAGTTGATGAGCAGTTACACTCGGACCTC GTCGACATGCATT |
| PIK3CA#4 | TGAAATCTGGTCGCCTCATTTGCTCAACTTACACTCGGACCTCGTC GACATGCATT |
| PIK3CA#5 | CTTGCCTATTCAGGTGCTTCAAATACATCCTTACACTCGGACCTCG TCGACATGCATT |

| | |
|---|---|
| PIK3CA#6 | CCATAGCCTGTTCAGGTTTGATTGGAGGCTTACACTCGGACCTCGT CGACATGCATT |
| PIK3CA#7 | TCGGGGATAGTTACACAATAGTGTCTGTGACTTTACACTCGGACCT CGTCGACATGCATT |
| PIK3CA#8 | GAGAGGATCTCGTGTAGAAATTGCTTTGAGCTTTACACTCGGACCT CGTCGACATGCATT |
| PIK3CA#9 | CTAGTCTGTTACTCAGTCCTGCGTGGGAATTACACTCGGACCTCGT CGACATGCATT |
| PIK3CA#10 | GCTAAATCCTGCTTCTCGGGATACAGACTTACACTCGGACCTCGTC GACATGCATT |
| PIK3CA#11 | AAGCCATTTTTCCAGATACTAGAGTGTCTGTGTTACACTCGGACCT CGTCGACATGCATT |
| PIK3CA#12 | AGTGTTCCTCTTTAGCACCCTTTCGGCTTACACTCGGACCTCGTCG ACATGCATT |
| PIK3CB#1 | AGTGCTTCTTCTTCACTCTTCCCTAATGTTACACTCGGACCTCGTC GACATGCATT |
| PIK3CB#2 | TATATCTTTGACTGATGTGAGTTCAGGAAGCCTTACACTCGGACCT CGTCGACATGCATT |
| PIK3CB#3 | CTGCAGCAGCCACATTGCTACTGTTCTTACACTCGGACCTCGTCGA CATGCATT |
| PIK3CB#4 | CTGAATGTCAGCAATTGTTTCAGAGGTGCTCATTACACTCGGACCT CGTCGACATGCATT |
| PIK3CB#5 | ACTTCAATGAGGCCAGAGCGATCTCCTTTACACTCGGACCTCGTC GACATGCATT |
| PIK3CB#6 | CCTTCCCTTTGGCTCTGTTTAACTTCACTTACACTCGGACCTCGTC GACATGCATT |
| PIK3CB#7 | CCCTATCCTCCGATTACCAAGTGCTCTTACACTCGGACCTCGTCGA |

| | |
|---|---|
| | CATGCATT |
| PIK3CB#8 | ATTGAGAAAGTTCTTCATCACTCATCTGTCGCTTACACTCGGACCTCGTCGACATGCATT |
| PIK3CB#9 | CATTAGCACTATCACTGCTTGCAATCTCTTACACTCGGACCTCGTCGACATGCATT |
| PIK3CB#10 | CTGCCTTTTCAATAATCTTATCGAAGGGAGGGTTACACTCGGACCTCGTCGACATGCATT |
| PIK3CB#11 | AGGATAATGCACTTTTCCAGCTTTCCTGATTTACACTCGGACCTCGTCGACATGCATT |
| PIK3CB#12 | TACCTCTGAGCTTACGATGGTTTTACACAGGTTACACTCGGACCTCGTCGACATGCATT |
| PIK3CD#1 | TGCACCAGCTGCAGCAGGTACTGGAATTACACTCGGACCTCGTCGACATGCATT |
| PIK3CD#2 | TTGATGGCGAAGGAGCCTACGTGGCAATTTACACTCGGACCTCGTCGACATGCATT |
| PIK3CD#3 | GGGAAGCTGAAGTCTAGCAGCTCCAGGTTACACTCGGACCTCGTCGACATGCATT |
| PIK3CD#4 | CCACATCCTCATGCTTGTTCCACTTGTTACACTCGGACCTCGTCGACATGCATT |
| PIK3CD#5 | CTCCGGGAAGTGCTCCTGGACTTCATTTACACTCGGACCTCGTCGACATGCATT |
| PIK3CD#6 | CACACCAGGTCCTTCTCGTGCTCATATTACACTCGGACCTCGTCGACATGCATT |
| PIK3CD#7 | CTCGGTGACATGCACACACTCGCTGTTTACACTCGGACCTCGTCGACATGCATT |
| PIK3CD#8 | GTCCTTGTAGTCAAACAGCATGAGGTTGGCTTACACTCGGACCTCGTCGACATGCATT |

| | |
|---|---|
| PIK3CD#9 | CTTCTTGGTGGAGCGAGCCTTCTTGGCTTACACTCGGACCTCGTCGACATGCATT |
| PIK3CD#10 | TCTCGATCACGGCGTACAGCGCAAAGCTTACACTCGGACCTCGTCGACATGCATT |
| PIK3CD#11 | CTTCATCCGCTCGTCGGCGTTCACTTTTACACTCGGACCTCGTCGACATGCATT |
| PIK3CD#12 | AGGCTTCTTCGCAGGAATGGGAGGTGGTTTACACTCGGACCTCGTCGACATGCATT |
| PIK3R1#1 | ACACAATGCTTTACTTCGCCGTCCACTTACACTCGGACCTCGTCGACATGCATT |
| PIK3R1#2 | CTACAGAGCAGGCATAGCAGCCCTGTTTACACTCGGACCTCGTCGACATGCATT |
| PIK3R1#3 | CTCGCTTCCCTCGCAACAGGTTTTCAGTTACACTCGGACCTCGTCGACATGCATT |
| PIK3R1#4 | TTGTTTCGGTTGCTGCTTCCAACATTCCATTACACTCGGACCTCGTCGACATGCATT |
| PIK3R1#5 | TCTTCTCATCATGATGGGGCAAATCTTCATTACACTCGGACCTCGTCGACATGCATT |
| PIK3R1#6 | ATCTTCCACCAGTGAATATTGGTCTTCAGTGTTACACTCGGACCTCGTCGACATGCATT |
| PIK3R1#7 | TTCATTGCCCAACCACTCGTTCAACTTCTTACACTCGGACCTCGTCGACATGCATT |
| PIK3R1#8 | TTGCCGAACACCTTTTTGAGTCAACCACATTTACACTCGGACCTCGTCGACATGCATT |
| PIK3R1#9 | AGTATTGGTCTCTCGTCTTTCTCAGCTGGTTACACTCGGACCTCGTCGACATGCATT |
| PIK3R1#10 | GGTCTGGTTTAATGCTGTTCATACGTTTGTCATTACACTCGGACCTC |

| | |
|---|---|
| | GTCGACATGCATT |
| PIK3R1#11 | TCGATACTCAGCTGCCTGCTTCTTCATTACACTCGGACCTCGTCGA CATGCATT |
| PIK3R1#12 | TCATTTGGATTTCCTGGGATGTGCGGGTATTTACACTCGGACCTCG TCGACATGCATT |

**Table S4. The sequences of the primary probes and FLAP probes used for FISH–FRET.**

| Probe ID | Sequence (5′-3′) |
|---|---|
| Cy5-tagged FLAP | Cy5-AATGCATGTCGACGAGGTCCGAGTGTAA-Cy5 |
| Cy3-tagged FLAP | Cy3-CTTATAGGGCATGGATGCTAGAAGCTGG-Cy3 |
| PIK3CA_cap#1 | TCAGCGGCCGCAGCGGCGGCACCGGAACTCCAGCTTCTAGCATCC ATGCCCTATAAG |
| PIK3CA_cap#2 | CAGTCCCAGCGGCCGCGGCAGCAGCACCAGCTTCTAGCATCCATG CCCTATAAG |
| PIK3CA_cap#3 | AGCTGCCCGGCCGGGCCCGAGCCCTGCCTCCAGCTTCTAGCATCC ATGCCCTATAAG |
| PIK3CA_tail#1 | GCTAGGAACAGCAAGACTGGGAAGAGGTCTTTACACTCGGACCT CGTCGACATGCATT |
| PIK3CA_tail#2 | CTGATGAGCATAAACAGTAAGTGCAGTCACTTACACTCGGACCTC GTCGACATGCATT |
| PIK3CA_tail#3 | CAGCGTGATGTTACAGACTGCAGAGTTAAAGTTACACTCGGACCT CGTCGACATGCATT |
| PIK3CB_cap#1 | ACAGAGCACGCGCGCGCCGCCGCCGAACCAGCTTCTAGCATCCAT GCCCTATAAG |
| PIK3CB_cap#2 | TCCCCACTGCCATGGCCCGCTCCGCCGCACCAGCTTCTAGCATCCA TGCCCTATAAG |
| PIK3CB_cap#3 | ATCCCTGCCTCTCTCGATCACCTCCCGCCTCCAGCTTCTAGCATCC |

| | |
|---|---|
| | ATGCCCTATAAG |
| PIK3CB_tail#1 | GTCACTTACCCAGGAATCTAACAAACGTTACACTCGGACCTCGTCGACATGCATT |
| PIK3CB_tail#2 | CCAGTTCTCTCTGGTGATGCTGATGCTCCTCTTACACTCGGACCTCGTCGACATGCATT |
| PIK3CB_tail#3 | CTTGAGGTAATGGATACCCCAATTACCCTGATTACACTCGGACCTCGTCGACATGCATT |
| PIK3CD_cap#1 | GCTCGGAGCGACTGCGCTGGGCGCGAGTCCAGCTTCTAGCATCCATGCCCTATAAG |
| PIK3CD_cap#2 | TTATCGTCCCGGCGCAGCTGGCAGGGCTCCAGCTTCTAGCATCCATGCCCTATAAG |
| PIK3CD_cap#3 | GATGCTTTAGAATCAATGAGTGTCATCCCGCCAGCTTCTAGCATCCATGCCCTATAAG |
| PIK3CD_tail#1 | CTAACTCCAGCCGGAGCGGGGTTTGTCTTTTACACTCGGACCTCGTCGACATGCATT |
| PIK3CD_tail#2 | GATAGGTCAGAAAGTGTATCCGCTACAATTTACACTCGGACCTCGTCGACATGCATT |
| PIK3CD_tail#3 | GTCGCCGCGCGGTTTCCCTTCGCAGATGTTACACTCGGACCTCGTCGACATGCATT |
| PIK3R1_cap#1 | GCTGCTTCCTCCAACTCCGCTCCAGCTGCTCCAGCTTCTAGCATCCATGCCCTATAAG |
| PIK3R1_cap#2 | GTGCTTGCCGTCTCCGACAGCTAGCCTCCAGCTTCTAGCATCCATGCCCTATAAG |
| PIK3R1_cap#3 | TTCCGACCGGCCTCCAGCCCGGCTGCGACCAGCTTCTAGCATCCATGCCCTATAAG |
| PIK3R1_tail#1 | AACTGACCGTGACATCCTCCCTCTCGTCAATTACACTCGGACCTCGTCGACATGCATT |

| | |
|---|---|
| PIK3R1_tail#2 | CACTACCCCCACCCCCCACCCCAGTTGCCTTTTACACTCGGACCTCGTCGACATGCATT |
| PIK3R1_tail#3 | CATAGCTCCATTGAATCTATAACTTGAACGTTACACTCGGACCTCGTCGACATGCATT |
